# ZNF432 stimulates PARylation and inhibits DNA resection to balance PARPi sensitivity and resistance

**DOI:** 10.1093/nar/gkad791

**Published:** 2023-10-12

**Authors:** Julia O’Sullivan, Charu Kothari, Marie-Christine Caron, Jean-Philippe Gagné, Zhigang Jin, Louis Nonfoux, Adèle Beneyton, Yan Coulombe, Mélissa Thomas, Nurgul Atalay, X Wei Meng, Larissa Milano, Dominique Jean, François-Michel Boisvert, Scott H Kaufmann, Michael J Hendzel, Jean-Yves Masson, Guy G Poirier

**Affiliations:** CHU de Québec Research Center, HDQ Pavilion, Oncology Division, Laval University Cancer Research Center, 9 McMahon, Québec City, QCG1R 3S3, Canada; CHU de Québec Research Center, CHUL Pavilion, Oncology Division, Laval University Cancer Research Center, 2705 Boulevard Laurier, Québec City, QCG1V 4G2, Canada; CHU de Québec Research Center, HDQ Pavilion, Oncology Division, Laval University Cancer Research Center, 9 McMahon, Québec City, QCG1R 3S3, Canada; CHU de Québec Research Center, CHUL Pavilion, Oncology Division, Laval University Cancer Research Center, 2705 Boulevard Laurier, Québec City, QCG1V 4G2, Canada; Department of Oncology, Faculty of Medicine and Dentistry, University of Alberta, 11560 University Avenue, Edmonton, Alberta T6G 1Z2, Canada; CHU de Québec Research Center, CHUL Pavilion, Oncology Division, Laval University Cancer Research Center, 2705 Boulevard Laurier, Québec City, QCG1V 4G2, Canada; CHU de Québec Research Center, HDQ Pavilion, Oncology Division, Laval University Cancer Research Center, 9 McMahon, Québec City, QCG1R 3S3, Canada; CHU de Québec Research Center, HDQ Pavilion, Oncology Division, Laval University Cancer Research Center, 9 McMahon, Québec City, QCG1R 3S3, Canada; CHU de Québec Research Center, HDQ Pavilion, Oncology Division, Laval University Cancer Research Center, 9 McMahon, Québec City, QCG1R 3S3, Canada; CHU de Québec Research Center, HDQ Pavilion, Oncology Division, Laval University Cancer Research Center, 9 McMahon, Québec City, QCG1R 3S3, Canada; Division of Oncology Research, Mayo Clinic, Rochester, MN 55905, USA; CHU de Québec Research Center, HDQ Pavilion, Oncology Division, Laval University Cancer Research Center, 9 McMahon, Québec City, QCG1R 3S3, Canada; Department of Immunology and Cell Biology, Université de Sherbrooke, Sherbrooke, Québec J1E 4K8, Canada; Department of Immunology and Cell Biology, Université de Sherbrooke, Sherbrooke, Québec J1E 4K8, Canada; Division of Oncology Research, Mayo Clinic, Rochester, MN 55905, USA; Department of Oncology, Faculty of Medicine and Dentistry, University of Alberta, 11560 University Avenue, Edmonton, Alberta T6G 1Z2, Canada; CHU de Québec Research Center, HDQ Pavilion, Oncology Division, Laval University Cancer Research Center, 9 McMahon, Québec City, QCG1R 3S3, Canada; CHU de Québec Research Center, CHUL Pavilion, Oncology Division, Laval University Cancer Research Center, 2705 Boulevard Laurier, Québec City, QCG1V 4G2, Canada

## Abstract

Zinc finger (ZNF) motifs are some of the most frequently occurring domains in the human genome. It was only recently that ZNF proteins emerged as key regulators of genome integrity in mammalian cells. In this study, we report a new role for the Krüppel-type ZNF-containing protein ZNF432 as a novel poly(ADP-ribose) (PAR) *reader* that regulates the DNA damage response. We show that ZNF432 is recruited to DNA lesions via DNA- and PAR-dependent mechanisms. Remarkably, ZNF432 stimulates PARP-1 activity *in vitro* and *in cellulo*. Knockdown of ZNF432 inhibits phospho-DNA-PKcs and increases RAD51 foci formation following irradiation. Moreover, purified ZNF432 preferentially binds single-stranded DNA and impairs EXO1-mediated DNA resection. Consequently, the loss of ZNF432 in a cellular system leads to resistance to PARP inhibitors while its overexpression results in sensitivity. Taken together, our results support the emerging concept that ZNF-containing proteins can modulate PARylation, which can be embodied by the pivotal role of ZNF432 to finely balance the outcome of PARPi response by regulating homologous recombination.

## Graphical Abstract

**Graphical Abstract:**
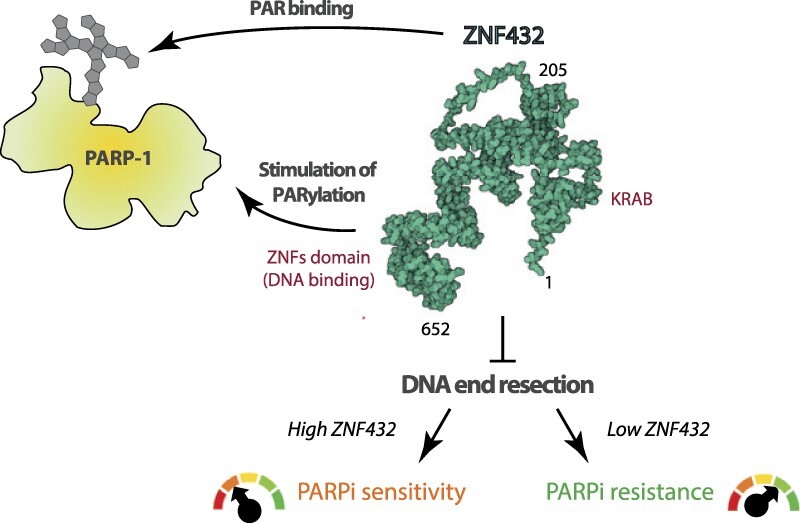

## Introduction

Zinc finger proteins (ZFPs) constitute the most abundant class of proteins in the human genome. They present a large diversity in terms of structure and functions. There are more than 40 types of zinc finger structures annotated in UniProt (1). The most frequently occurring sub-classes of zinc finger domains are C2H2, CCHC, PHD and RING domains. The classical C2H2 domain of 28–30 aa includes a β-hairpin (antiparallel β-sheet consisting of two β-strands), followed by an α-helix, which form a left-handed ββα structure (2). Once considered functioning exclusively as transcription factors, ZFPs emerged more than two decades ago as factors involved in the coordination of the cellular DNA damage response (DDR). For example, the BRCA1-associated and KRAB (Krüppel-associated box)-containing protein ZBRK1/ZNF350 and other members of the KRAB family were characterized as repressors coupling transcription with the DNA repair process (3). The KRAB-containing C2H2-ZNFs (KRAB ZFPs) show a typical domain architecture defined by the presence of a DNA-binding domain made up of between 4 and over 30 zinc finger motifs and a KRAB domain located near the amino terminus of the protein (4). Notably, a network of several KRAB ZFPs is connected by KAP1 (KRAB-associated protein 1), a molecular scaffold protein that acts as a corepressor and mediates protein-protein interactions (5). KAP1 is phosphorylated by ATM, ATR, and DNA-PKcs in response to DNA damage and colocalizes with hallmark DNA repair proteins such as 53BP1, γH2AX, and BRCA1 (6). Although there was a clear connection between gene silencing and KRAB ZFPs in DDR pathways, more recent studies revealed that a much wider variety of ZFPs, including several non-KRAB ZFPs, are recruited to DNA lesions and may play a role as effectors of the DDR (7). Interestingly, a systematic analysis of factors localized to damaged chromatin revealed a poly(ADP-ribose) polymerase-1 (PARP-1)-dependent recruitment dynamics for many ZFPs (8). Recent studies have identified numerous ZFPs that orchestrate the DDR and pathways related to genome integrity (reviewed in (9)). For example, C2H2-types ZFPs such as ZNF280C/ZPET and ZNF830 have been respectively characterized as an homologous recombination (HR) repressor and HR promoting factor by modulating DNA end resection (10,11) while ZNF281 and ZNF384 mediate DDR by facilitating the recruitment of non-homologous end-joining (NHEJ) factors to DNA damage sites (12,13). ZFPs are now seen as global genome maintenance regulators whose action is not limited to the regulation of gene expression. Through their multiple interactions with DNA repair factors, ZFPs contribute to finely balancing the use of distinct DNA repair systems.

Through a combination of a wide variety of different topologies and structures, ZFPs are highly adaptable and able to interact with DNA, RNA, poly-ADP-ribose (PAR), and other proteins (14–16). Owing to the development of high-throughput mass spectrometry technologies, proteome-wide analysis pinpointed ZFPs as a protein family which is overrepresented in PAR-associated protein datasets (17,18). PAR, as a biopolymer, has intrinsic characteristics that makes it a distinct post-translational modification (PTM). In addition to being *bona fide* substrates of PARPs via covalent PARylation of specific amino acid residues, proteins can also bind PAR in a non-covalent fashion. The latter referred to as PAR *readers*, which can be relocated to sites of DNA damage and have their functions reprogrammed, as has been shown for ZBTB24 and other ZFPs (12,19,20). A genome-wide screen of proteins involved in DNA double-strand break (DSB) repair by HR also identified a surprisingly large number of uncharacterized ZFPs as putative components of the DNA damage response (21). Because PARP-1-dependent PARylation has major implications in the DDR, including the regulation of DSBs that represents one of the most lethal types of DNA damage (22), we recently directed our attention to a group of ZFPs found to possess an affinity for PAR. Using a protein microarray that covers most of the human proteome, Kang and colleagues identified a group of C2H2-type ZFPs that could be characterized as PAR *readers* (23). Based on this observation, we explored the possibility that some of these ZFPs could act as effectors of HR-based DNA repair through which PARylation could play a potential regulatory role. We report that the C2H2-type ZNF and KRAB-containing protein ZNF432 is a novel HR repressor involved in the cellular response to DNA damage and PARPi.

## Materials and methods

### Antibodies, reagents, oligonucleotides and siRNA

The antibodies used in this study and their working dilutions are listed in Supplementary Table S1. Key reagents or resources are listed in Supplementary Table S2. Pre-designed SMARTpool that combined four gene-specific siRNA was purchased from Horizon Discovery. Sequences of siRNAs are provided in Supplementary Table S3. The sequence of γ-^32^P-labeled DNA oligonucleotides for gel retardation assays are indicated in Supplementary Table S4. qPCR primer sequences used in ER-*Asi*SI resection assay are listed in Supplementary Table S5. pEGFP-C1-ZNF432 (GFP-ZNF432) and pFastBac1-GST-ZNF432-10XHis plasmids were constructed by subcloning of ZNF432 coding sequence from pcDNA3.1-ZNF432(NM_014650.2)-DYK (GenScript, OHu20285D).

### Cell culture, transfection and virus production

Human cell lines HEK293T (CRL-11268) and HeLa (CCL2) were purchased from ATCC. The ER-*Asi*SI U2OS cell line was maintained in phenol red-free DMEM media supplemented with 10% charcoal-stripped FBS (Sigma), 50 IU of penicillin, 50 μg/ml streptomycin and 1 μg/ml puromycin. The EJ5-GFP HEK293T cell line was maintained in DMEM supplemented with 10% FBS, 50 IU of penicillin, 50 μg/ml streptomycin and 0.5 μg/ml puromycin. U2OS CRISPR-ZNF432 knockout cells (termed KD due to the remaining levels of ZNF432) were generated by Ubigene Biosciences. HEK293T PARP-1 KO cells were previously generated by our group (22). Sf9 insect cells (B82501) were purchased from Thermo Fisher Scientific. All cell lines were free of mycoplasma and were grown in DMEM supplemented with 10% FBS, 50 IU of penicillin and 50 μg/ml streptomycin unless specified. COV362 cells knockout for 53BP1 were generated previously (24). COV362 cell lines were grown in RPMI supplemented with 10% FBS, 50 IU of penicillin and 50 μg/ml streptomycin. Sf9 cells were grown in Gibco Grace's insect medium supplemented with 10% FBS and 50 IU of penicillin and 50 μg/ml streptomycin for 2D culture and in Gibco Sf-900 II SFM for suspension culture.

DNA transfections were carried out using Effectene and Invitrogen Lipofectamine 2000 transfection reagents according to the manufacturer's instructions. Lipofectamine RNAiMAX was used as per the manufacturer's protocol with some modifications to transfect 62.5 nM siRNA. In brief, the siRNAs and Lipofectamine RNAiMAX were diluted separately in Opti-MEM media without FBS or antibiotics and were incubated at room temperature (RT) for 5 min. Both solutions were mixed (lipids and siRNAs) with gentle pipetting and were again incubated at RT for 20 min. During the incubation time, the media of the cells was changed with Opti-MEM media containing 10%FBS. After incubation, the lipids:siRNAs mixture was added to the cells. The transfection medium was replaced after 24 h of transfection for DMEM complete media. The cells were analyzed 48 to 72 h post-transfection.

ZNF432 overexpressing baculoviruses were prepared by transfecting pFastBac1-GST-ZNF432-10XHis bacmid in Sf9 cells using Cellfectin reagent (Gibco) as per the manufacturer's instructions.

### Affinity-purification coupled to mass spectrometry (AP-MS)

The plasmid vectors coding for GFP and GFP-ZNF432 were transfected in HEK293T cells for 24 h in 150 mm petri dishes. Two washes with ice-cold Phosphate Buffered Saline (PBS) were carried out prior to harvesting the cells with a disposable cells scraper. All the affinity-purification steps were carried out at 4°C unless mentioned otherwise. Three ml/plate of CHAPS-containing buffer [40 mM HEPES pH 7.5 (Millipore Sigma, H3784), 0.3% (w/v) CHAPS (Roche, 10810118001), 150 mM NaCl (Thermo Fisher Scientific, BP3581) and supplemented with Complete protease inhibitor cocktail (Roche, 11697498001)] were used to prepare whole cell extracts. Cell extracts were kept on ice and lysed for an additional 20 min on a rotating device. Cell extracts were centrifuged for 5 min at 3400 rpm to remove cellular debris. Affinity-purification experiments were performed using Dynabeads magnetic beads covalently coupled to Protein G (Invitrogen). The Dynabeads were washed once with 2 ml of PBS and coated with 15 μg of mouse monoclonal anti-GFP antibody (Roche). The beads were then washed 3 times with 2 ml of lysis buffer and added to the whole cell extract for a 2 h incubation with gentle rotation. Samples were then washed 3 times with 1 volume of lysis buffer for 10 min. Beads were resuspended in a 75 mM ammonium bicarbonate solution pH 8.0 (Millipore Sigma, A6141). Proteins were first reduced with 15 mM DTT (Millipore Sigma, I-6125) for 20 min at RT and alkylated with 30 mM iodoacetamide (Millipore Sigma, D0632) under the same conditions but protected from light. Protein complexes were directly digested on-beads by the addition of 1 μg of a Trypsin/Lys-C mixture. Peptides were isolated on C18 resin tips according to the manufacturer's instructions (Thermo Fisher Scientific) and dried to completion in a SpeedVac evaporator (Thermo Scientific Savant).

### LC-MS/MS analysis

Peptides were separated using a Dionex Ultimate 3000 nanoHPLC system. Ten μL of sample (a total of 2 μg) in 1% (vol/vol) formic acid were loaded with a constant flow of 4 μL/min onto an Acclaim PepMap100 C18 column (0.3 mm id x 5 mm, Dionex Corporation). After trap enrichment, peptides were eluted onto an EasySpray PepMap C18 nano column (75 μm x 50 cm, Dionex Corporation) with a linear gradient of 5–35% solvent B (90% acetonitrile with 0.1% formic acid) over 240 min with a constant flow of 200 nL/min. The HPLC system was coupled to a Q Exactive Orbitrap mass spectrometer (Thermo Fisher Scientific) via an EasySpray source. The spray voltage was set to 2.0 kV and the temperature of the column set to 40°C. Full scan MS survey spectra (m/z 350–1600) in profile mode were acquired in the Orbitrap with a resolution of 70000 after accumulation of 1000000 ions. The ten most intense peptide ions from the preview scan in the Orbitrap were fragmented by collision-induced dissociation (normalized collision energy 35% and resolution of 17500) after the accumulation of 50000 ions. Maximal filling times were 250 ms for the full scans and 60 ms for the MS/MS scans. Precursor ion charge state screening was enabled and all unassigned charge states as well as singly, 7, and 8 charged species were rejected. The dynamic exclusion list was restricted to a maximum of 500 entries with a maximum retention period of 40 secs and a relative mass window of 10 ppm. The lock mass option was enabled for survey scans to improve mass accuracy. Data were acquired using the Xcalibur software.

### Mass spectrometry data analysis

Mass spectra data (*.raw files) were searched using Byonic version 3.3.3 (Protein Metrics, USA) against the *Homo sapiens* reference proteome (canonical & isoforms, 75 069 entries, UniProt) with a static modification of carbamidomethylation on Cys (+57.0215 Da) and the following variable modifications: carbamidomethylation on His, Lys and peptide N-terminal (+57.0215 Da), oxidation of Met (+15.9949 Da), formation of pyro-Glu from N-terminal Glu and Gln residues (-18.0105 Da for N-Term Glu and -17.0265 Da for N-term Gln), deamidation of Asn and Gln (+0.9840 Da), amidation of Glu and Asp (-0.9840 Da) and N-terminal peptide acetylation (+42.0105 Da). Fully specific tryptic cleavage was specified, and a maximum of two missed cleavages was allowed. The search tolerance was set to 7 ppm for the precursor ions and 20 ppm for the fragment ions. A false discovery rate (FDR) of 1% or less was estimated using concatenated forward–reverse database search. A minimum Log Prob (*i.e*. -Log base_10_ of the protein p-value) of 1.3 (p-value ≤ 0.05) was selected as the protein identification cutoff value. Decoys protein IDs and common contaminants were filtered out of the final dataset.

### ZNF432 protein-protein interaction network analysis

The search tool for retrieval of interacting genes (STRING) (https://string-db.org) database (25), which integrates both known and predicted PPIs, was used to predict functional interactions among ZNF432-associated proteins identified by AP-MS. Active interaction sources, including text mining, experiments, databases, and co-expression (limited to *Homo sapiens*) and an interaction score > 0.4 were applied to construct the network. The DAVID online gene annotation tool (26) was used to identify DDR-associated proteins and chromatin remodelers (based on Gene Ontology and The UniProt Consortium assignments).

### Immunofluorescence

The HeLa cells were grown on coverslips. For the detection of RAD51, RPA, phosphorylated DNA-PKcs (pDNA-PKcs), 53BP1, RIF1, and γH2AX foci, cells were either untreated or treated with 5 Gy X-ray irradiation generated in a CellRad irradiator (Precision X-Ray Irradiation). For RAD51 analysis, cells were incubated for 3 h post-irradiation, fixed with 4% paraformaldehyde (PFA, w/v) (J.T. Baker, S898-04) for 10 min at RT, washed 3 times with PBS and fixed again with ice-cold methanol for 5 min. After final fixation, cells were washed 3 times with PBS and permeabilized with 0.2% Triton X-100 (Millipore Sigma, T8787) in PBS for 5 min at RT. Following 3 washes with PBS, cells were quenched with 0.1% sodium borohydride in PBS for 5 min at RT.

For staining RPA, cells were incubated for 1 h post-irradiation. Cells were treated with 10 μM EdU for 15 min and subjected to *in situ* fractionation on ice for 10 min using sequential extraction with two different buffers. Pre-extraction buffer 1A [(10 mM PIPES, pH 7.0, 300 mM sucrose (Fisher, S5-3) 100 mM NaCl, 3 mM MgCl_2_ (Millipore Sigma, 208337) and 0.5% Triton X-100)] followed by pre-extraction buffer 2A [10 mM Tris-HCl pH 7.5, 10 mM NaCl, 3 mM MgCl_2,_ 1% Nonidet *P*-40 (Millipore Sigma, I3021) and 0.5% sodium deoxycholate (Millipore Sigma, D6750)].

For staining with phospho-RPA, cells were either untreated or treated with 5Gy irradiation and released for 1 h. After 45 min of release EdU was added in accordance with the Thermofisher Click-it protocol. Prior to fixation, cells were subjected to *in situ* fractionation on ice for 10 min using sequential extraction with two different buffers. Pre-extraction buffer 1 (10 mM PIPES, pH 7.0, 300 mM sucrose, 100 mM NaCl, 3 mM MgCl_2_ and 0.5% Triton-X100) followed by pre-extraction buffer 2 (10 mM Tris pH 7.5, 10 mM NaCl, 3 mM MgCl_2_, 1% Nonidet *P*-40 and 0.5% sodium deoxycholate). The cells were then fixed with 4% paraformaldehyde in PBS for 25 min on ice. Following PFA removal the coverslips were washed three times with PBS, and the cells were permeabilized with PBS containing 0.5% Triton X-100 for 15 min and washed three times with PBS. Following permeabilisation the Click-it protocol was continued according to ThermoFisher's instructions. The cells were then blocked in PBS containing 10% FBS for 1 h and incubated with the primary antibody (pRPA 1:500) diluted in the blocking buffer for 2 h at room temperature. Coverslips were washed three times with PBS before 1 h incubation with the appropriate secondary antibody (1:1000) conjugated to a fluorophore again in blocking buffer. The coverslips were rinsed again twice with PBS, then incubated in (1:1000) PBS-Hoechst solution for 5 min, then washed twice with PBS. Coverslips were mounted onto slides with ProLong Gold antifade mounting media (Thermo Fisher Scientific).

For pDNA-PKcs, 53BP1, RIF1, and γH2AX analysis, cells were incubated for 1 h post-irradiation. pDNA-Pkcs experiments were also treated with 10 μM EdU for 15 min after irradiation. Cells from all experiments (except RAD51) were fixed with 4% PFA for 20 min at RT and permeabilized with 0.2% Triton X-100 in PBS for 20 min at RT. Cells used for analysis of RPA and pDNA-Pkcs were treated with the EdU Click-iT reaction to mark S-phase cells according to the manufacturer's protocol (Thermo Fisher Scientific). Cells from each experiment were washed 3X with 1X PBS and were incubated with blocking buffer (10% goat serum, 1% BSA in PBS) for 1 h, subsequently the cells were incubated with primary antibodies in PBS supplemented with 1% BSA for 2 h at RT. Geminin was used as a S-phase marker in RAD51, 53BP1, RIF1, and γH2AX experiments. Following primary incubation coverslips were washed three times with PBS before 1 h incubation with the appropriate Alexa conjugated secondary antibodies in PBS supplemented with 1% BSA. The coverslips were rinsed twice with PBS then incubated with DAPI (1:1000) solution for 5 min to stain DNA. Finally the coverslips were washed twice more with PBS prior to mounting onto slides with ProLong Gold antifade mounting media (Thermo Fisher Scientific).

Z-stack images were acquired on a ZEISS Celldiscoverer 7 microscope using a 25 × water immersion objective, then deconvolved and analyzed for RAD51 foci formation with ZEN 3.2 black edition software (Zeiss). The number of RAD51 foci per Geminin-positive cells was scored using automatic spot counting by ZEN software and validated manually. Data from three independent trials were analyzed for outliers using the ROUT method (Q = 1.0%) in GraphPad Prism and the remaining were reported in a scatter dot plot. Horizontal lines on the plots designate the median values.

### *Cell cycle identification by Click-it* EdU Cell Proliferation Kit and Alexa Fluor 647 dye

Cell cycle identification was performed using the Click-iT EdU Cell Proliferation Kit (ThermoFisher). 15 min prior to fixation, cells were incubated with 10 μM EdU in cell media. Following the incubation, the media containing EdU was removed, and the cells were washed twice with PBS, then fixed with 4% PFA for 25 min. The PFA was removed, and the coverslips were washed 3 times with PBS. The cells were permeabilized with PBS containing 0.5% Triton X-100 for 15 min, then washed three times with PBS. 100 μl of the Click-it reaction cocktail (containing an Alexa Fluor 647 dye and prepared according to the manufacturer's instructions) was placed on each coverslip. The plate was incubated at room temperature for 30 min in the dark. The click-it reaction cocktail was removed and the coverslips washed twice with PBS. From this point, the antibody-specific immunofluorescence protocol was resumed, though the plate was protected from light throughout the rest of the staining.

### BrdU/ssDNA assays

HeLa cells were incubated with 10 μM BrdU (Millipore Sigma) for 16 h, this was followed by 3 h incubation after 5 Gy irradiation. Cells were subjected to *in situ* fractionation on ice for 10 min using sequential extraction with two different buffers. Pre-extraction buffer 1B (10 mM PIPES pH 7.0, 300 mM sucrose, 100 mM NaCl, 3 mM MgCl_2_, 1 mM EGTA and 0.5% Triton X-100) and followed by pre-extraction buffer 2B (10 mM Tris pH 7.5, 10 mM NaCl, 3 mM MgCl_2,_ 1% Tween 20 and 0.5% sodium deoxycholate). The coverslips were washed twice with PBS prior to fixation with 4% PFA (w/v) for 20 min at 4°C. Cells were washed with PBS and then fixed with methanol (5 min at -20°C). Cells were washed with PBS, permeabilized with 0.5% Triton X-100 and incubated with blocking buffer (PBS supplemented with 3%BSA for 1 h). Cells were incubated overnight at 4°C with anti-BrdU and anti-PCNA antibodies in blocking buffer. Unbound primary antibody was removed by washing with PBS at RT followed by incubation with secondary antibodies in PBS supplemented with 1% BSA for 1 h at RT. Following DAPI counterstaining, coverslips were mounted onto slides with ProLong Gold antifade mounting media. The integrated intensity of BrdU foci per nucleus was analysed as described previously (27).

### Laser micro-irradiation

Wild-type HEK293T or PARP-1 KO cells were transfected with either GFP or GFP-ZNF432 coding plasmids. To evaluate the PAR-dependent recruitment of GFP-ZNF432 at DNA damage sites, cells were treated with 1 μM PARP inhibitor BMN673 for 16 h prior to analysis. A 37°C pre-heated stage with 5% CO_2_ perfusion was used for the time-lapse on a LSM 510 META NLO laser-scanning confocal microscope at 40X magnification (Zeiss). Localized DNA damage was generated along a defined region across the nucleus of a single living cell by using two-photon excitation of the Hoechst 33342 dye, generated with a near-infrared 750-nm titanium:sapphire laser line (Chameleon Ultra II, Coherent). The laser output was set to 2% with 10 iterations. For each condition, 32 images were collected at a 15 secs interval. The average accumulation ± SEM of GFP-ZNF432 was plotted using a minimum of ten recruitment kinetic profiles from three independent experiments.

For the recruitment of EXO1-GFP by laser-induced DNA damage, U2OS cells were seeded onto 35-mm fluorodishes (World Precision Instruments, Inc.) and transfected with siCTRL or siZNF432 small interfering RNA (siRNA) using RNAiMAX (Invitrogen) for a final concentration of 50 nM twice (0 h and 24 h). After 48 h, cells were transfected with 1 μg GFP-EXO1 using lipofectamine 2000 transfection reagent. For time-lapse microscopy, cells were micro-irradiated using point bleach mode for 200 ms with a 405 nm UV-laser (100% output) at the following settings: format 512 × 512 pixels, scan speed 100 Hz, mode bidirectional, zoom 2X, 16 bit image depth. To monitor the recruitment of GFP-EXO1 to laser-induced DNA damage sites, cells were imaged every 10 sec for 3 min on a Leica TCS SP5 II confocal microscope driven by Leica LAS AF software. The fluorescence intensity of GFP-EXO1 at DNA damage sites relative to an unirradiated area was quantified and plotted over time. Data show the mean relative fluorescence intensity ± S.E. of approximately 145 cells per condition from at least 4 independent experiments.

### GFP-trap pull-down assay

Hela cells were transfected overnight with GFP or GFP-ZNF432 coding plasmids in a 10 cm dish. The following day, cells were exposed to 10 Gy irradiation and allowed to recover for 15 min. After incubation, the cells were trypsinized and collected by centrifugation at 2000 x g for 5 min. The cells were then lysed with 1 ml of ice-cold lysis buffer (50 mM Tris-HCL pH 7.5, 150 mM NaCl, 0.5% NP-40, 1 mM PMSF, 0.019 UIT/ml aprotinin, 1 μg/ml leupeptin, 5 mM NaF and 1 mM Na_3_VO_4_) and incubated on ice for 30 min. The cells were sonicated for 15 min with a 30 s on/off cycle. Subsequently, cells were centrifuged at 4°C for 30 min at maximum speed. The supernatant was added to 25 μL of GFP-trap agarose beads (ChromoTek) and supplemented with 25 U/ml Benzonase endonucleases (Millipore Sigma, E8263) and 2.5 mM MgCl_2_. The mixture was incubated at 4°C for 4 h on a rotating device. The beads were spun down at 2500 x g for 2 min and were washed 3 times with the lysis buffer. The supernatant was discarded, and the beads were resuspended in Laemmli sample buffer for SDS-PAGE and Western blot analysis.

### ZNF432 protein purification

Sf9 insect cells (3L at 2 × 10^6^ cells/ml) were infected with a baculovirus designed for the expression of GST-ZNF432-His and were incubated at 27ºC in a shaking incubator for 48 h. For the KRAB and ZNFs fragments, *E. coli* BL21RP were transformed with a plasmid designed for the expression of GST-ZNF432KRAB-His or GST-ZNF432ZNFs-His. Six liters of bacteria were then grown at 37°C until they reached an OD between 0.4 and 0.6 before being induced with 0.1 mM IPTG and incubated O/N at 16°C. Cells were harvested by centrifugation and the pellet was frozen on dry ice. Cells were lysed in Buffer 1 (175 mM KPO_4_ pH 8.0, 300 mM KCl, 1 mM DTT and protease inhibitors) containing 0.05% Triton X-100 followed by 5 times 30 secs on/off cycles of sonication on ice. The cell lysate was incubated with 1 mM MgCl_2_ and 15 U/ml Benzonase endonucleases at 4°C for 45 min followed by centrifugation at 35 000 rpm for 45 min. The soluble cell lysate was incubated with GST-Sepharose beads for 90 min at 4°C with gentle rotation. The beads were washed twice with Buffer 1 followed by incubation with HSP buffer (Buffer 1 supplemented with 5 mM ATP and 15 mM MgCl_2_) for 45 min at 4°C. GST-Sepharose beads were washed twice with Buffer 1 supplemented with 200 mM KCl and once with P5 buffer (50 mM NaHPO_4_ pH 7.0, 500 mM NaCl, 10% glycerol, 0.05% Triton X-100 and 5 mM imidazole). Following washing, the beads were incubated in P5 buffer containing 25 mM glutathione (pH between 7.0 and 8.0) to release the protein from the beads. Both the beads and the glutathione elution were cleaved with PreScission protease (60 U/ml, GE Healthcare Life Sciences), overnight at 4°C in P5 buffer. The beads were then incubated again for 1 h at 4ºC in P5 containing 25 mM glutathione to release the protein left on the beads. The supernatant was collected and combined with the other fractions. The supernatant mixture was incubated with Talon beads for 1 h at 4°C on gentle rotation. The beads were washed 3X with P5 buffer followed by 2 times with P30 buffer (P5 buffer with a final concentration of 30 mM imidazole) and transferred into a 1.5 ml tube. Proteins were eluted twice with P500 buffer (P5 buffer containing 500 mM imidazole) and dialyzed for 1 h at 4°C against the storage buffer (20 mM Tris-HCl pH 7.4, 200 mM NaCl, 10% glycerol and 1 mM DTT) and stored in aliquots at − 80°C.

### Non covalent PAR-binding assay

Proteins were slot-blotted on a 0.2 μm pore size nitrocellulose membrane (Bio-Rad) using a Bio-Dot microfiltration apparatus (Bio-Rad). Ten pmoles of proteins diluted in 200 μL TBS (20 mM Tris-HCl pH 7.5, 150 mM NaCl) were applied to the vacuum manifold sample template. Calf thymus histone H1 (Calbiochem, 382150) was used as a positive control PAR *reader* in addition to DNAse I (Roche, 10104159001) and BSA (Millipore Sigma, P0914) as negative control PAR-binding proteins. Following complete aspiration of the protein samples, the nitrocellulose membrane was rinsed three times in 50 ml TBS-T (20 mM Tris-HCl pH 7.5, 150 mM NaCl, 0.01% Tween-20). The membrane was incubated with TBS-T containing 100 nM PAR purified by dihydroxyboronyl Bio-Rex (DHBB) chromatography according to Shah *et al.*(28). The membranes were extensively washed with TBS-T and blocked with a PBS-MT solution (1X PBS supplemented with 5% milk and 0.1% Tween-20) for 1 h. The membrane was then incubated overnight with the anti-PAR monoclonal antibody clone 10H (Tulip Biolabs). The membrane was extensively washed with PSM-MT and incubated with an anti-mouse peroxidase-conjugated secondary antibody (Jackson Immuno Research) for 30 min. Following incubation, the membrane was washed 3 times with 1X PBS containing 0.1% Tween-20 and revealed with the Western Lightning Plus ECL chemiluminescence substrate (PerkinElmer). SYPRO Ruby protein blot stain was used according to the manufacturer's protocol (Bio-Rad) to assess the efficiency of protein transfer to the nitrocellulose membrane.

### PARP-1 *in vitro* activity assays

PARP-1 activity assays were performed in 50 μL of reaction buffer [100 mM Tris-HCl pH 8.0, 10 mM MgCl_2_,10 mM DTT, 10% (v/v) ethanol, and 25 μg/ml calf thymus activated DNA (Millipore-Sigma) containing 1 pmole of PARP-1 and increasing amounts (1, 2 and 5 pmoles) of highly purified full-length ZNF432 (aa 1–652), the N-terminal ZNF432 KRAB fragment (aa 1–205), the C-terminal ZNF432 ZNFs region (aa 205–652), HPF1 (Tulip Biolabs) and bovine serum albumin (BSA, Millipore-Sigma) as a control. Proteins were pre-incubated for 10 min at RT to promote complex formation. Proteins were then incubated for 5 min at 37°C with 1 mM NAD^+^ before stopping the reaction with 4x laemmli sample buffer (Bio-Rad) containing 5% β-mercaptoethanol. To analyse the specificity of PAR linkages, the PARP-1 automodification reactions were stopped with 1.6 mM PARP-1 inhibitor BMN673 and subjected to 1M hydroxylamine hydrolysis (Millipore-Sigma). PARG treatments were performed by supplementing the reaction products with 1 μg of a fully-active 60 kDa PARG fragment prepared as in Whang Z. *et al.* PloS One 2014 (29). Reaction products were resolved by 4–12% linear gradient SDS-PAGE (Bio-Rad) and transferred onto a 0.2 μm nitrocellulose membrane. PAR was revealed by Western blot using the anti-MAR/PAR antibody E6F6A (Cell Signaling Technology) or the anti-PAR antibody 96–10 (produced in-house); PARP-1 was detected with the mouse monoclonal anti-PARP-1 antibody clone C2-10 (produced in-house) while full-length ZNF432 and the KRAB fragment were detected with a rabbit antipeptide (Novus Biologicals) directed towards the N-terminal region of human ZNF432 (aa 71–120).

### DNA-binding assays

γ-^32^P-labeled DNA oligonucleotides (100 nM, sequence of oligonucleotides are indicated in Supplementary Table S4) were added to MOPS (3-(N-morpholino)propanesulfonic acid) binding buffer (25 mM MOPS pH 7.0, 60mM KCL, 40 nM CaCl_2,_ 0.2% Tween, 2mM DTT)_._ The mixture was incubated at 37ºC for 5 min. An increasing amount of purified ZNF432 (1, 2, 4, and 6 nM) was added to the DNA-buffer mix and was incubated at 37ºC for 30 min. The total reaction volume was 10 μL. After incubation, the protein–DNA complexes were fixed with 0.2% (v/v) glutaraldehyde for 20 min at 37°C. The reactions were subjected to electrophoresis at 150V for 1.5 h at 4°C on an 8% TBE acrylamide gel and γ-^32^P-labeled DNA was visualized by autoradiography.

For DNA binding assays without fixation for Kd calculations, γ-^32^P-labeled DNA oligonucleotides (100 nM Supplementary Table S4) were added to MOPS (3-(N-morpholino)propanesulfonic acid) binding buffer (25 mM MOPS pH 7.0, 60 mM KCL, 1 mM CaCl_2,_ 0.2% Tween, 2mM DTT)_._ The mixture was incubated at 37ºC for 5 min. Increasing concentrations of purified full-length ZNF432 or its KRAB or ZNF domains (1, 2, 4, and 6 nM) were added to the DNA-buffer mix and incubated at 37ºC for 30 min. The reactions were subjected to electrophoresis at 150V for 2 h at 4°C on an 8% TBE acrylamide gel and γ-^32^P-labeled DNA was visualized by autoradiography. For K_d_ calculations, or each concentration of protein, a mean of the triplicate assays was used. The percentage of bound protein to the DNA was plotted in GraphPad Prism depending on the concentration to obtain the B_max_ and K_d_ for each protein and each DNA substrate. The Scatchard plots were obtained by plotting the B_max_ and B_max_/K_d_ for each condition.

### DNA resection assays

Purified EXO1 or the DNA resection machinery were purified as described previously (22,30). Assays were performed with pUC18 DNA linearized with *Kpn*I and then 3′ labeled with [α-^32^P] ATP and terminal deoxynucleotidyltransferase. Reactions were conducted using 50 nM of substrate in standard buffer (20 mM Na-HEPES pH 7.5, 0.1 mM DTT, 0.05% Triton X-100 and 100 μg/ml BSA). Two mM ATP and 5 mM MgCl_2_ were added to the reaction buffer at the beginning of the reactions. In the experiment involving PAR, 200 nM purified PAR was added to the reaction 5 min prior to the addition of ZNF432. The reactions were initiated on ice by adding ZNF432 (10, 20, 30, and 40 nM) and transferred to 37°C for 5 min. After incubation, 6.5 nM of purified EXO1 was added and the reaction was incubated for 45 min at 37°C. Reactions were followed by proteinase K treatment for 1 h at 37°C. Products were analyzed on a 1% native agarose gel. Gels were dried on DE81 paper (Whatman), and signals were detected by autoradiography. Densitometric analyses were performed using the FLA-5100 phosphor-imager (Fujifilm) and quantified using the Image Reader FLA-5000 v1.0 software.

### Induction of DSBs in i265 U2OS cells

Inducible 265 U2OS (i265) cells were grown in DMEM supplemented with 10% FBS. Shield1 (1 μM) and 4-OHT (1 μM) were added into medium to incubate with i265 U2OS cells for an accumulation of nuclear mCherry-FokI to generate DSBs. Cells were pre-extracted twice with RPA extraction buffer (20 mM HEPES, pH 7.9, 50 mM NaCl, 300 mM sucrose, 1mM EDTA, 3 mM MgCl_2_, 0.5% Triton X-100) for 10 mins before being fixed at 1 h of mCherry-FokI to induce DSBs. Cells were fixed with 4.0% paraformaldehyde in PBS, pH 7.5 for 12 mins at room temperature. Immunostaining was performed as previously described. A Carl Zeiss (Imager Z.1) microscope equipped with epifluorescence optics was used to collect the images for inducible i265 (i265) cells. Images were captured with a 40 × objective (NA = 1.4) connected to a SensiCam QE charge-coupled device camera (PCO TECH, Inc.), using Metamorph 7.0 software (Universal Imaging Corp.). For intensity analyses in i265 cells, mCherry-FokI positive cells were chosen randomly and the elliptical tool (ImageJ software from the NIH) was used to select a region of interest (ROI) on the mCherry-FokI focus for which the integrated fluorescence intensity was measured. A separate region outside of the cell was measured to determine background (BG). The relative integrated intensity ratios [ROI-BG)/BG] were plotted after normalization of the control average to 1.0. Briefly, cells were pre-extracted twice with RPA extraction buffer (20 mM HEPES, pH 7.9, 50 mM NaCl, 300 mM sucrose, 1mM EDTA, 3 mM MgCl_2_, 0.5% Triton X-100) for 5  mins before being fixed at 6–8 h after IR or restriction enzyme induced DSBs. Cells were fixed with 4.0% paraformaldehyde in PBS for 10  mins at room temperature. Immunostaining was performed using Rabbit anti-PAR (96–10, 1/1000 dilution, produced in-house), mouse anti-PARP-1 (clone C2-10 (1/1000 dilution, produced in-house) and γ-H2AX (1/1000 dilution, Active motif) as primary antibodies. Species specific conjugated with Alexa-488 or Cy5 were used as secondary antibodies.

### NHEJ in cellulo reporter assay

HEK293T cells stably expressing the EJ5-GFP reporter (a kind gift from Jeremy Stark, City of Hope, Duarte, USA) were obtained and were seeded onto polylysine-coated coverslips and transfected twice, 24 h apart, with the indicated siRNAs. Twenty-four hours after the second transfection, cells were transfected with pCAG.I-Sce1 plasmid using TransIT -293 (Mirus). Eight hours post-transfection, when indicated, cells were incubated with 3 μM NU77441 (DNA-PKcs inhibitor, Selleck Chemicals) or with vehicle (DMSO) for 48 h. Cells were fixed 48 h post-transfection with 4% paraformaldehyde for 20 min followed by washes with PBS and DAPI counterstaining. The percentage of GFP positive cells was assayed by fluorescence microscopy (3000 cells were counted based on DAPI nuclear staining). The percentage was expressed as fold-change normalized to the control siRNA or DMSO conditions.

### ER-*Asi*S1 resection assay

The percentage of resection adjacent to a specific DSB1(Chr 1: 89231183) was measured as described in (31) with some modifications. The primer pairs for ‘DSB1’ are across *Bsr*G1 restriction sites. Briefly, ER-*Asi*SI U2OS cells were transfected twice, 24 h apart, with the indicated siRNAs. Forty-eight hours after the second transfection, cells were treated with 300 nM of hydroxytamoxifen (4-OHT) (Sigma) for 4 h to allow the nuclear translocation of *Asi*SI and the induction of DSBs. Cells were collected and genomic DNA was purified using QIAGEN DNeasy kit. For each sample, 140 ng of extracted DNA was subjected to an RNAseH treatment for 20 min, and digested in 30 μL with 20 Units of *Bsr*gI HF (New England Biolabs), or mock digested (no enzyme) at 37°C overnight. Samples were heat inactivated at 65°C, and analyzed by qPCR. Digested or mock-digested samples were used as a template for qPCR performed using SYBR Green master mix. Primers used are listed in Supplementary Table S5. The percentage of ssDNA was calculated with the following equation: ssDNA% = 1/(2^(ΔCt−1)^ + 0.5) ×100, where ΔCt is calculated by subtracting the Ct obtained from mock-digested sample from the *Bsr*GI-digested sample.

### Monitoring in cellulo PAR intensity

Live cell imaging and micro-irradiation experiments were carried out with a Leica TCS SP5 II confocal microscope driven by Leica LAS AF software using a 63x/1.4 oil immersion objective. Briefly, HeLa cells seeded onto 35-mm fluorodishes (World Precision Instruments, Inc.) were co-transfected with 1 μg of PAR sensor pcDNA3-PBZ-mRuby2 (Addgene plasmid # 110650) (32) and 1 μg of peGFP or peGFP-ZNF432 using Lipofectamine 2000 transfection reagent (Invitrogen). The next day, cells were micro-irradiated in located spots in the nucleus for 100 ms using a 405 nm UV-laser at the following settings: format 512 × 512 pixels, scan speed 100 Hz, mode bidirectional, zoom 2X. To monitor the recruitment of the PAR sensor (PBZ-mRuby) to laser-induced DNA damage sites, cells were micro-irradiated and imaged every 10 sec for 5 min, after which fluorescence intensity of PBZ-mRuby at DNA damage sites relative to an unirradiated nuclear area was quantified and plotted over time. Kinetic curves were obtained by averaging the relative fluorescence intensity and error bars show the SEM. All results are from at least 3 independent experiments (total n > 95 cells/condition).

### Cell viability assay

AlamarBlue was chosen as the cell viability assay as used previously with PARP inhibitors (33,34). The U2OS wild-type (WT) or CRISPR-Cas9-generated ZNF432 knockdown (KD) cells were seeded in 96-well plates (7500 cells/well). After 16 h of seeding, cells were transfected with GFP or GFP-ZNF432 coding plasmids. Six hours post-transfection, the cells were treated with increasing concentrations of Talazoparib (BMN673) with a constant concentration of DMSO. For COV362, siRNA against ZNF432 was used. Post 16 h of siControl or siZNF432 transfection, the cells were treated with BMN673 or Olaparib (AZD2281). After 72 h of incubation with PARP inhibitors, the media was changed with 100 μL of culture media containing 10% AlamarBlue and the plates were incubated at 37ºC with 5% CO_2_ until the first appearance of pink color. The absorbance was measured at 560 nm (excitation) and 590 nm (emission) wavelengths. The experiments were performed in triplicates and repeated three times.

### Automated live-cell proliferation analysis

Twenty-four hours after siRNA transfection, cells were seeded in triplicates into 96-well cell culture plates at a density of 1000 COV362 or COV362 53BP1-/- cells per well. The media was supplemented 24 h later with DMSO (control vehicule) or a series of Olaparib (Selleckchem) concentrations. Plates were imaged every 6 h for 12 days with an IncuCyte SX5 live-cell analysis system (Sartorius) equipped with the 10× SX5 Green/Orange/NIR optical module. Cell number (determined by phase contrast) and proliferation growth data were generated using the Incucyte® Basic analysis software (2022B Rev 2). IC50 values were calculated with GraphPad Prism. Each experiment was performed in triplicates and repeated three times.

### Analysis of ovarian cancer datasets

Differences in the expression pattern of ZNF432 were analyzed in normal ovary tissue (*N* = 110) and ovarian cancer tissue (*N* = 1516) using data from gene expression databases of normal and tumor tissues (http://gent2.appex.kr/gent2/, accessed on January 2022). Furthermore, the cancer tissue samples were stratified into the most common subtypes of ovarian cancer (clear: 60, endometroid: 43, mucinous: 19 and serous: 247) to analyze the difference in the expression pattern of ZNF432. The significance analysis for the comparison of ZNF432 expression was performed using GraphPad Prism. For normal ovary tissue versus ovarian cancer tissue samples, an unpaired Student's *t*-test was used, whereas Kruskal–Wallis one-way ANOVA analysis of variance was used to determine the significance of the differences in ZNF432 expression patterns among different subtypes of ovarian cancer as compared to normal ovary tissue.

### FACS analysis of cell cycle

Cell cycle analysis was performed through flow cytometry with propidium iodide (PI) DNA staining. Cells were trypsinized, centrifuged and washed with PBS. The cells were centrifuged again and resuspended in 300 μl of PBS and fixed with 100% ethanol (EtOH) overnight. Prior to the flow cytometry the cells are stained with a mixture of PI (100 μg/ml) and RNAse (50 μg/ml) for 30 min in the dark on ice. The cell cycle distribution was performed on a BD Accuri C6 Plus flow cytometer.

### Statistical analyses

All data are representative of three or more independent experiments. GraphPad Prism was used to do the statistical analyses. For the analysis of foci, significance was determined applying Mann–Whitney *U*-test. For the NHEJ in cellulo reporter assay, the lac array intensity analysis and the ER-*Asi*S1 resection assay, significance was calculated by the unpaired *t*-test. *P ≤ 0.05, **P ≤ 0.01, ***P ≤ 0.001, ****P ≤ 0.0001.

## Results

### ZNF432 is a novel DDR factor that contributes to HR-mediated DSB repair

**Figure 1.**
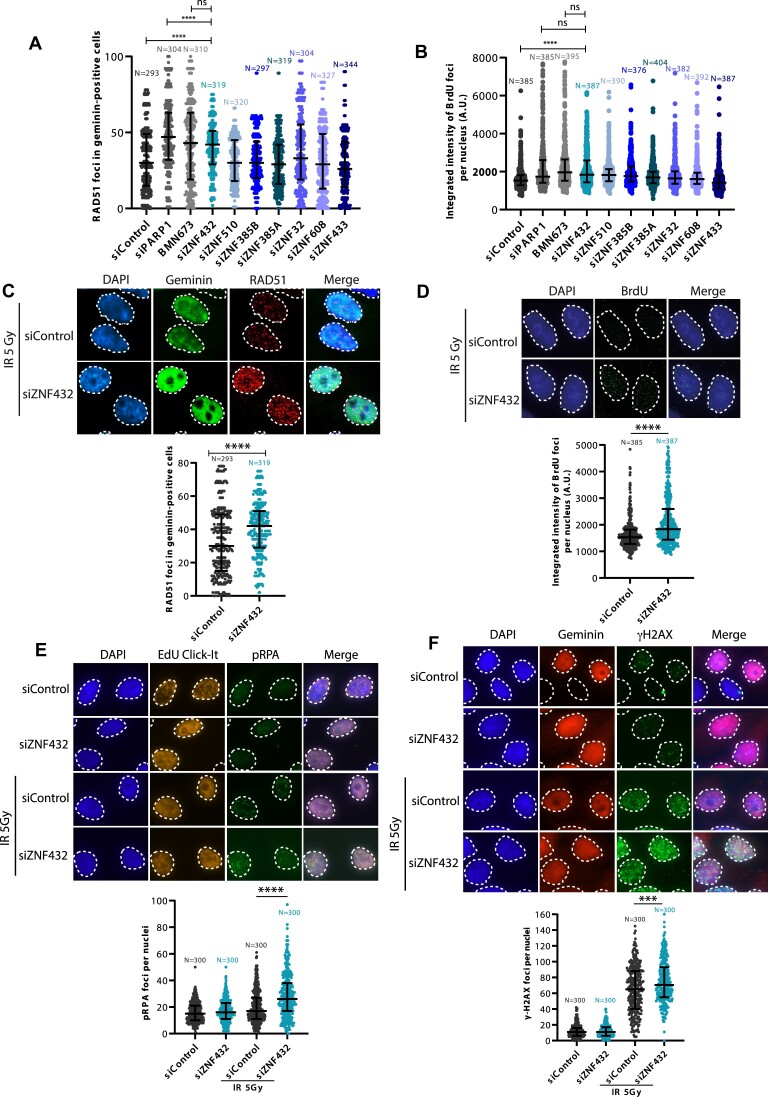
Identification of potential resection regulators in a subset of ZFPs identified by proteome-wide microarray analysis of PAR readers. HeLa cells transfected with control or gene-specific siRNAs against 7 selected ZFPs were irradiated (5 Gy) and screened for RAD51 (**A**) and BrdU (**B**) foci formation 3 h post-irradiation. siRNA-mediated PARP-1 knockdown and inhibition with 1 μM BMN673 for 16 h were taken as positive controls. The data presented in the graphs are the median of three independent experiments. The effects of ZNF432 depletion on RAD51 (**C**), BrdU foci formation (**D**), pRPA (**E**) and γ-H2AX (**F**), are shown. S-phase cells were identified by selecting Geminin for RAD51 and γ-H2AX foci analysis or EdU Click-iT labeling of EdU incorporated into cells for pRPA foci analysis. The error bars represent ± s.d. *P ≤ 0.05, **P ≤ 0.01, ***P ≤ 0.001, ****P ≤ 0.0001 (Mann–Whitney *U*-test).

PARylation has been recognized to coordinate the recruitment of several critical proteins that participate in the DDR and guide the DNA repair pathways. To find PAR readers affecting DSB repair, we searched for PAR readers in a data set of non-covalent PAR-binding proteins identified in a proteome-wide microarray-based screen (23). The C2H2-type ZNF protein domain was identified as the most statistically significant ontology term within PAR readers. We selected a group of 7 ZFPs identified as PAR-binding proteins from the microarray for an evaluation of their potential role in HR. The early steps of HR involve the resection of DNA (5′–3′ exonucleolytic degradation) leading to a stretch of 3′-ended single-stranded DNA (ssDNA) which is then repaired by the RAD51 recombinase using the sister chromatid as a DNA template (35). siRNA-induced gene silencing for each ZFP target was performed in a pre-screen experiment to analyze their effect on RAD51 (Figure 1A and C) and BrdU foci (Figure 1B and D) formation after irradiation using immunofluorescence analysis. In the BrdU assay, resected DNA exposes BrdU-incorporated regions of single-stranded DNA which serves as a readout for DNA resection. The KRAB-ZFP ZNF432 showed the most significant effect, increasing both RAD51 and BrdU foci formation (Figure 1A, B). We also validated our findings of increased RAD51 foci and BrdU foci upon ZNF432 knockdown using siRNA against ZNF432 (Supplementary Figure S1A and C). The efficacy of siZNF432 in downregulating ZNF432 expression in different cell lines is depicted in Supplementary Figure S1A. We also aimed to validate our findings in a knockout context. To do this, we used Ubigene CRISPR-Cas9 technology (https://www.ubigene.us) to knockout ZNF432 in U2OS cells. Unfortunately, they only reached partial depletion of the gene product with this approach. However, a specific clone, called F1, showed a significant decrease in the expression of endogenous ZNF432 (Supplementary Figure S1B). Henceforth, the F1 clone is referred to as ZNF432 KD cells due to partial inhibition of ZNF432. The CRISPR-Cas9-generated ZNF432 KD cells also displayed increased RAD51 foci and BrdU foci (Supplementary Figure S1D, and E). ZNF432 was therefore selected for further characterization.

To corroborate the increase of DNA resection using BrdU immunofluorescence levels as a surrogate, we also measured the accumulation of phosphorylated replication protein A (RPA) in ZNF432 knockdown cells. DNA resection results in a long stretch of 3′-ssDNA, which is expeditiously coated by RPA to prevent degradation and the formation of secondary structures. Phosphorylation of RPA (pRPA) inhibits further resection of DNA by the resection machinery and hence could be marked as the endpoint of resection (36). Consistently, ZNF432 reduction notably increased the formation of pRPA foci indicating heightened generation of ssDNA by the resection machinery (Figure 1E). Histone H2AX phosphorylation (γ-H2AX) serves as a quantifiable event to evaluate the early cellular response for the induction of DNA DSBs. More γ-H2AX foci were observed in cells treated with siZNF432 as compared to siControl cells, further suggesting that depletion of ZNF432 causes defects in DSB repair (Figure 1F).

**Figure 2.**
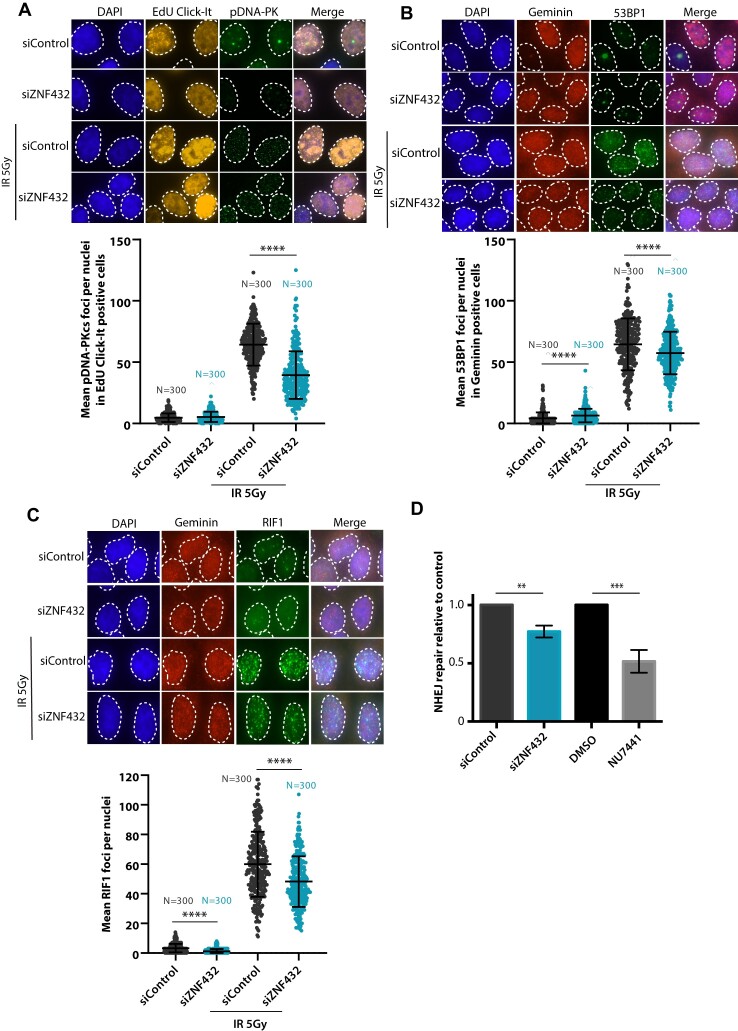
Loss of ZNF432 decreases NHEJ. The effect of ZNF432 knockdown in HeLa cells following IR-induced damage on (**A**) phospho-DNA-PKcs, (**B**) 53BP1 and (**C**) RIF1 foci formation. The cells were selected for S-phase by using EdU Click-iT (A) or S-G2-phase cells with Geminin staining (B, C). The error bars indicate ± s.d. of three independent experiments. *P ≤ 0.05, **P ≤ 0.01, ***P ≤ 0.001, ****P ≤ 0.0001 (Mann–Whitney *U*-test). (**D**) The attenuation of ZNF432 decreases NHEJ. EJ5-GFP HEK293T cells were transfected with pCAG.I-Sce1 and the percentage of GFP-positive cells was evaluated 48 h later by fluorescence microscopy. The siControl and DMSO conditions were normalized to 1.0. The error bars represent s.d. from three independent experiments. **P ≤ 0.01, *** *P* ≤ 0.001 (Student's *t*-test).

To determine if ZNF432 affects NHEJ, the other main pathway of DSB repair, we measured the accumulation of phospho-DNA-PKcs foci in ZNF432 knockdown cells following IR. Phosphorylation of DNA-PKcs (pDNA-PKcs) correlates with activation of NHEJ. Using the Click-iT EdU detection reagent to select S-phase specific cells, we observed a significant decrease in the accumulation of pDNA-PKcs foci in S-phase cells depleted of ZNF432 (Figure 2A). This suggests that ZNF432 knockdown decreases NHEJ-mediated DSB repair, which was corroborated using HEK293T cells harboring a NHEJ reporter cassette (Figure 2D). We also quantified the formation of IR-induced 53BP1 and RIF1 foci in the context of ZNF432 knockdown. 53BP1 and RIF1 are both negative regulators of resection that promote DSB repair by NHEJ over HR. Using geminin as a marker of cells in the S/G2-phase, we observed a decrease of 53BP1 and RIF1 foci in ZNF432 knockdown cells compared to control cells (Figure 2B and C). Similar results were obtained for 53BP1 (Supplementary Figure S2A) and RIF1 (Supplementary Figure S2B) in CRISPR-Cas9-generated U2OS ZNF432 KD cells. A reduction in the accumulation of HR repressors such as 53BP1 and RIF1 following ZNF432 depletion also suggests that the absence of ZNF432 tilts the DSB repair balance towards the HR pathway. Furthermore, we observed a shift in the cell cycle upon downregulation of ZNF432 facilitating HR. Flow cytometry analysis of ZNF432 KD cells revealed an increase in the proportion of cells in the G2 phase and a reduction of cells in the G1 phase (Supplementary Figure S2C). Globally, these results suggest that ZNF432 acts as a novel DDR factor and point toward a role in HR-mediated DSB repair.

### ZNF432 relocalizes to sites of DNA damage via DNA- and PAR-dependent mechanisms

**Figure 3.**
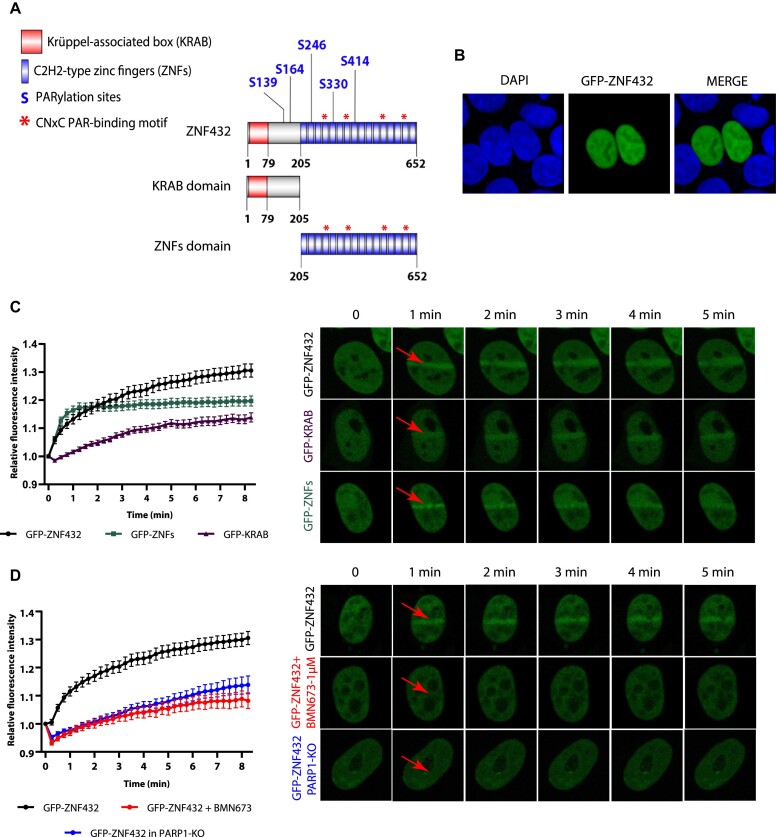
Recruitment of ZNF432 to DNA damage depends on PARP-1 and PAR synthesis. (**A**) ZNF432 protein structure. (**B**) Cellular localization of ZNF432. Green—GFP-ZNF432 full length, blue—DNA (Hoechst). (**C**) Graph and representative images showing the recruitment of full-length GFP-ZNF432 and its subdomains GFP-ZNFs and GFP-KRAB in HEK293T cells following the damage induced by laser-microirradiation. (**D**) Effect of inhibition of PAR (1 μM BMN673 for 16 h) and loss of PARP-1 (PARP-1 KO) on recruitment of ZNF432 (GFP-ZNF432) to DNA lesions in HEK293T cells, following the damage induced by laser-microirradiation. The error bars represent ± SEM of three independent experiments.

ZNF432 bears a Krüppel-associated box (KRAB) domain and a zinc finger (ZNF) domain consisting of 16 repeats (Figure 3A). Five serine PARylation sites were identified in ZNF432, of which three are located in ZNF domains (18,37,38,39). ZNFs 4, 7, 12 and 15 contain the recently identified CNxC PAR-binding motif common to several C2H2-type ZFPs (23). As a first step towards the systematic functional characterization of ZNF432 in DSB repair and HR, we expressed GFP-ZNF432 as a fusion protein in 293T cells and determined its subcellular localization. Similar to other KRAB-ZFPs, GFP-ZNF432 is a nuclear protein excluded from the nucleolus (Figure 3B). It has been demonstrated that the KRAB domain targets proteins in the nucleoplasm through interaction with TRIM28/KAP1 (KRAB-associated protein 1) while ZNFs target proteins in the whole nucleus uniformly. The cooperative activities of KAP1-KRAB-ZNFs result in the precise nucleoplasmic, but not nucleolar localization of KRAB-ZFPs (40). Using laser-induced micro-irradiation (micro-IR) of sub-nuclear regions, we showed that GFP-ZNF432 responds to DNA damage by relocating to damage sites. In order to determine which domain is responsible for the recruitment of ZNF432 to DNA lesions, GFP-KRAB (1–205) and GFP-ZNFs (205–652) fusion proteins were expressed in 293T cells and their recruitment was monitored using micro-IR. Both domains are expressed as nuclear proteins, an observation supported by the discovery that both KRAB and ZNFs possess nuclear localization activities (Figure 3C) (40). Both domains can be independently relocalized to sites of micro-IR but the recruitment of the region containing the ZNFs repeats is recruited with more intensity, especially during the earlier phases of the process (Figure 3C). This observation is consistent with the identification of specific ZNFs as PAR recognition modules essential to mediate the recruitment of CTCF (CCCTC-binding factor) to DNA lesions (41). Following this idea, the contribution of PAR-binding in the dynamics of GFP-ZNF432 recruitment to DNA damage sites was evaluated. The dynamics of GFP-ZNF432 recruitment under normal conditions were compared with those under PARP inhibition (PARPi) with BMN673 and in PARP-1 knockout cells. Both PARPi and PARP-1 depletion significantly delayed the accumulation of GFP-ZNF432 recruitment to DNA damage sites (Figure 3D). These results strongly suggest that the localization of GFP-ZNF432 to laser-induced DSBs is PAR-dependent.

### ZNF432 is a PAR reader that interacts with PARP-1 and chromatin remodeling proteins

**Figure 4.**
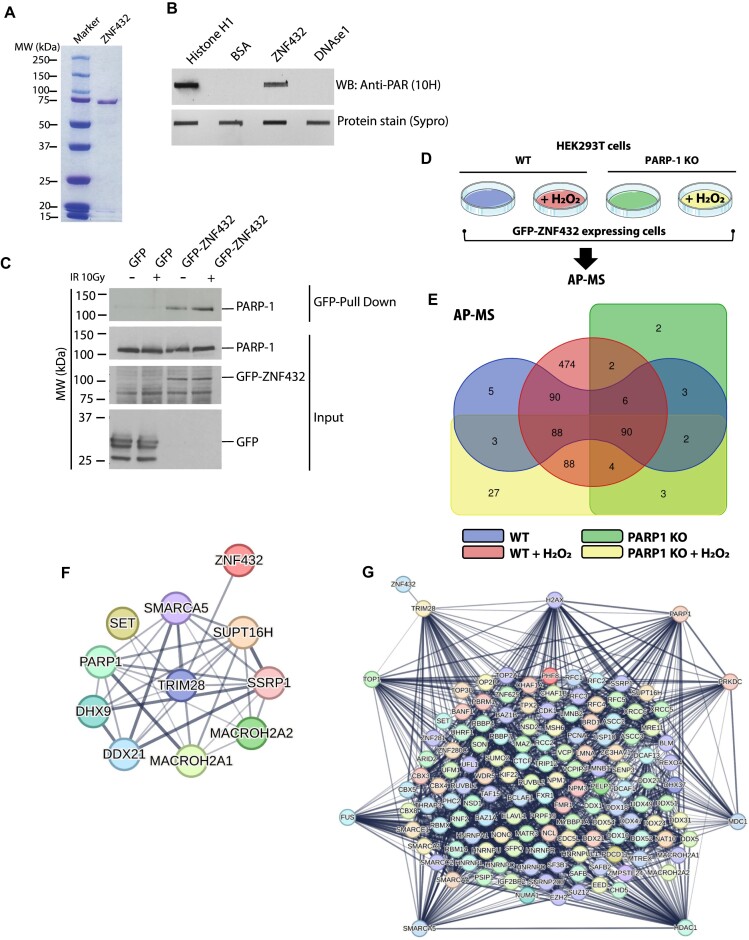
ZNF432 interacts with PAR and PARP-1. (**A**) Sodium dodecyl sulfate-polyacrylamide gel electrophoresis of purified human ZNF432 protein. (**B**) Overlay assay showing the affinity of ZNF432 for PAR *in vitro*. Five pmoles of recombinant human ZNF432 were slot-blotted on a nitrocellulose membrane and probed with 100 nM protein-free DHBB-purified PAR. Histone H1 and DNAse I were respectively used as positive and negative PAR binding controls. The presence of PAR bound to proteins was revealed by western blot (WB) with the anti-PAR antibody clone 10H. SyproRuby protein blot staining was used as a loading control. (**C**) GFP-ZNF432 interacts with endogenous PARP-1. (**D**) Experimental design for the identification of ZNF432 protein interaction networks by affinity-purification coupled to mass spectrometry (AP-MS). (**E**) Venn diagram of overlapped and unique ZNF432-associated proteins identified by AP-MS. The number of proteins in the overlapping and non overlapping area are shown. Whole cell extracts of wild-type (WT) and PARP-1 depleted HEK 293T cells (PARP-1 KO) were prepared following exposure to hydrogen peroxide (H_2_O_2_) or left untreated. Refer to Supplementary Table S6 for a complete listing of proteins in each area. (**F**) ZNF432 resides in a core protein complex in which TRIM28/KAP1 acts as a central hub. The interaction network (derived from the STRING-DB website) shows some of the most abundant ZNF432 interaction partners. (**G**) ZNF432 protein interaction network generated from 139 proteins assigned to DNA damage response and chromatin remodeling pathways. Nodes with high connectivity in the protein interaction network were isolated. The edges indicate both functional and physical protein associations. Line thickness indicates the strength of data support.

The relocation of DDR proteins to DNA lesions is often solely dependent on PAR-induced chromatin remodeling rather than PAR binding, a situation where DNA-binding proteins are more efficiently guided to exposed DNA (42). In order to further support the contribution of PAR in the dynamics of ZNF432 at DNA damage sites that we observed with laser-induced DSB recruitment assays, we performed a PAR overlay assay with purified ZNF432 (Figure 4A). Using free PAR isolated from automodified PARP-1, we showed that ZNF432 binds PAR *in vitro* (Figure 4B). GFP-trap pull-down of GFP-ZNF432 shows that PARP-1 resides in ZNF432 protein complexes (Figure 4C). The association of ZNF432 with PARP-1 was confirmed by affinity-purification coupled with mass spectrometry (AP-MS) under basal conditions and PARP-1 activation following hydrogen peroxide-induced DNA damage (Figure 4D). A total of 887 proteins co-isolated with ZNF432 were identified by LC–MS/MS (Figure 4E). In addition to several chromatin remodelers and DDR-associated factors, the KRAB-associated protein 1 (KAP1/TRIM28) was identified as one of the most abundant ZNF432-interacting protein in HEK 293T cells (refer to Supplementary Table S6 for a complete list of proteins co-eluted with ZNF432). The relative abundance of this prey suggests a robust promiscuous interaction between the protein partners. Indeed, KAP1 is a common partner of most KRAB-containing ZFPs (43). KAP1 acts as a scaffold for the transcription silencing machinery but is also a major substrate of ATM, in response to DNA DSBs, that promotes chromatin decondensation for efficient DNA repair (44,45). Using PARP-1-depleted cells, we observed that the KAP1-ZNF432 complex is stable and does not dissociate in the absence of PARP-1 nor after PARP-1 activation. Similarly, components of FACT complex (SSRP1 and SUPT16H (46)) or the nuclear oncogene SET (45) appear to form a stable complex with ZNF432 (Figure 4F). However, we do observe that hundreds of proteins are specifically identified in the ZNF432 protein interaction network after PARP-1 activation, including several DDR-associated proteins. Using the STRING protein-protein interaction database, we looked into the potential links between 139 ZNF432-interacting proteins with assigned functions in various DDR pathways. A very dense network of DDR-associated proteins was generated. We observed that a small number of highly connected nodes, corresponding to key mediators of DSB repair and chromatin remodeling, emerged from the network of interactors (KAP1/TRIM28, PARP-1, H2AX, PRKDC/DNA-PKcs, MDC1, HDAC1, SMARCA5, FUS and TOP1) (Figure 4G). A variety of proteins involved in the maintenance of genomic integrity are located within the network (*e.g*. BLM, MRE11, XRCC6/KU70, XRCC5/KU80, components of the polycomb repressive complex 2 (EED, EZH2 and SUZ12) (47)) or DDR-associated ZFPs such as CTCF (41,48), ZNF280C/ZPET (10) and ZNF281 (12) that all contributes to DNA DSB repair. These results suggest that assemblies of ZNF432 interacting proteins can be reshaped following PARP-1-induced PARylation in complex dynamics although a highly stable core protein complex exists. The characterization of ZNF432 as a PAR reader in PAR overlay assay and the identification of PARP-1 in ZNF432 protein complexes add further support to the idea that ZNF432 regulates the DDR through a PARP-1-dependent pathway.

### ZNF432 stimulates PARP-1 activity and modulates PARylation dynamics at DSBs

**Figure 5.**
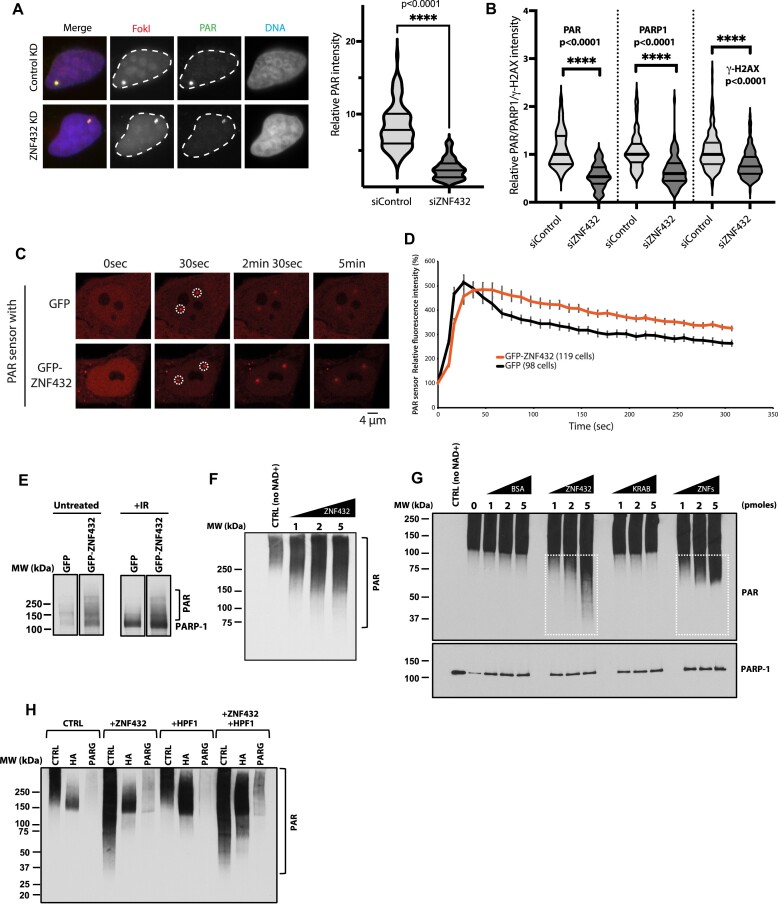
ZNF432 promotes PARP-1 assembly and activation. (**A**) Left: Knockdown of ZNF432 reduces PARylation at localized DSBs. i265 U2OS cells were transfected with control or ZNF432 siRNAs for 7 days before being analyzed at 1 h after DSB induction. The PAR signal is shown in green, while the DSB sites were marked by mCherry-FokI in red. Nuclei were stained by Hoechst 33342 in blue. Right: A violin plot displaying the quantification of relative PAR intensity in siControl or siZNF432 cells in the experimental setting of Figure 5A. The dotted line indicates the mean. The slide line below the mean labels the 1st quartile and the slide line above the mean demarcate the 3rd quartile, respectively. *P*-values were calculated by unpaired two-tailed Student's *t*-test as shown. (**B**) i265 U2OS cells transfected with siRNAs (control siRNA and ZNF432 smart pool) were processed at 1 h post-DSB induction for immunofluorescence. The violin plot represents the quantification of the relative intensity of PAR, PARP-1 and γ-H2AX foci on the array. (**C**) Overexpression of GFP-ZNF432 increase PARylation as measured with a PBZ-mRuby PAR sensor. (**D**) Monitoring of the mRuby-PAR relative fluorescence intensity over time. (**E**) ZNF432 stimulates PARP-1 activity. Overexpression of GFP-ZNF432 increases intracellular PAR levels under basal conditions and in IR-treated cells. Whole cell extracts of ZNF432-expressing cells were analyzed for the presence of PAR by western blot. (**F**) Western blot showing the *in vitro* automodification pattern of PARP-1 in the presence of full length human ZNF432 (1, 2 and 5 pmoles). PARP-1 was incubated with ZNF432, calf-thymus activated DNA and NAD+. Reaction products were resolved by SDS-PAGE and PAR polymers were identified by western blot. A polyclonal antibody against PAR (96–10) was used in both blots E and F to reveal the presence of PAR polymers. (**G**) The ZNFs region of ZNF432 stimulates PARP-1 activity. The Western blot shows the *in vitro* auto modification pattern of PARP-1 in the presence of bovine serum albumin (BSA, control), full length ZNF432 (1–652), the N-terminal fragment containing the KRAB domain (1–205) and the C-terminal domain containing the array of 16 ZNFs repeats (205–652). PAR polymers were revealed by Western blot analysis using a polyclonal antibody against MAR/PAR (E6F6A). (**H**) ZNF432 and HPF1 produce an additive stimulatory effect on PARP-1 activity. PARP-1 was auto modified *in vitro* in the presence of ZNF432, HPF1 and both proteins. Reaction products were subjected to 1M hydroxylamine (HA) hydrolysis to cleave Asp/Glu-linked ADP-ribosylation or PARG treatment to erase PAR polymers. PAR polymers were revealed by Western blot analysis using a polyclonal antibody against PAR (96–10).

The activation of PARP-1 is one of the earliest step in a series of events leading to DNA damage signaling and repair. Thus, we sought to determine if ZNF432 possesses regulatory activity for PARP-1 activation. Two strategies were used to monitor the enhanced ZNF432-dependent PARylation in cellulo. We exploited a localized DSB system where an array of Lac repressor DNA-binding sequences are inserted into U2OS cells. The system uses an inducible fusion protein containing the LacI DNA-binding domain fused to the Fok1 nuclease domain, which is responsible for the DSB formation, and mCherry which allows the identification of the DSB loci (49). Following ZNF432 knockdown, we observed that PARylation was decreased at the foci containing the array of DSBs (Figure 5A). Quantification of PAR, PARP-1, and phosphorylated histone H2AX levels revealed that PAR levels were most dramatically impacted, with a similar although slightly reduced reduction in PARP-1 accumulation. Phosphorylated histone H2AX levels were also significantly reduced, but not as dramatically as PARylation and PARP-1 accumulation (Figure 5B). ZNF432 overexpression led to an increase in PARylation after micro-irradiation as monitored with the PBZ-mRuby PAR sensor (Figure 5C and D). Consistent with this, overexpression of GFP-ZNF432 stimulated PARP-1 automodification (Figure 5E).

Using an *in vitro* PARP-1 automodification assays in the presence of ZNF432, NAD+, and an activating DNA substrate, PARylation was measured by Western blot to evaluate if ZNF432 affects PARylation profiles. We found that supplementation with ZNF432 stimulates the activity of PARP-1 (Figure 5F). We determined that the C-terminal region of ZNF432 encompassing the 16 ZNFs repeats stimulates PARP-1 activity, in contrast to the N-terminal KRAB domain-containing region (Figure 5G). Finally, we sought to determine whether the introduction of HPF1 modulates the stimulatory activity of PARP-1 observed in presence of ZNF432 (Figure 5H). The stimulatory effect of HPF1 was confirmed with 1M hydroxylamine treatments. Serine-linked ADP-ribose bonds are hydroxylamine-resistant while carboxyl-ester linkages established with glutamate and aspartate residues are sensitive to hydrolysis by hydroxylamine (50–53). As expected, most PAR synthesized in the presence of HPF1 is hydroxylamine-resistant while the stimulation of activity observed with ZNF432 does not result in a significant switch towards hydroxylamine-resistant ADP-ribose linkages, suggesting that PARylation occurs on glutamate and aspartate residues. However, we observed an additive stimulatory response of PARP-1 PARylation activity in the combined presence of HPF1 and ZNF432. In these assays, reaction products were treated with poly(ADP-ribose) glycohydrolase (PARG) to hydrolyze PAR and confirm the nature of the polymer. Overall, these results show that ZNF432 influence PARP-1 assembly and dynamics of PARylation at DNA damage sites.

### ZNF432 preferentially binds single-strand DNA and inhibits EXO1-mediated resection *in vitro*

**Figure 6.**
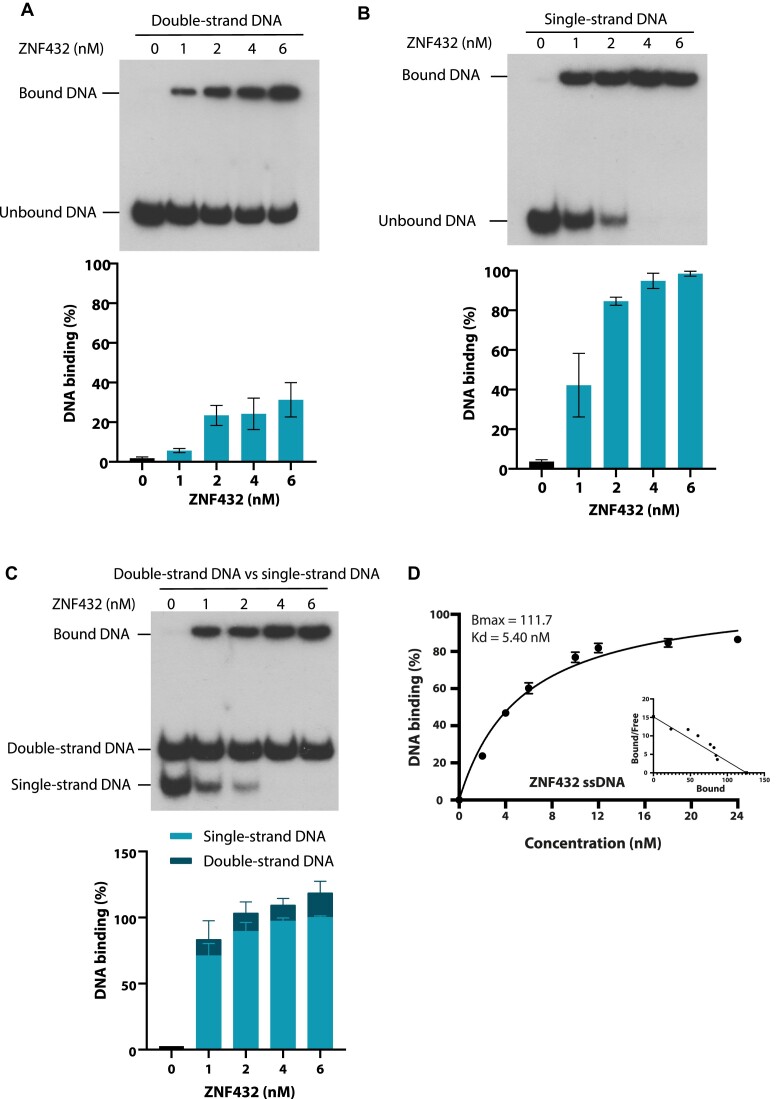
ZNF432 preferentially binds to single-stranded DNA. Purified ZNF432 DNA binding capabilities were monitored on the following labeled DNA substrates: (**A**) double-strand DNA, (**B**) single-strand DNA. (**C**) Competition bandshift assays using the single-strand DNA probe in competition with the double-strand DNA probe. All assays were done at 37ºC with glutaraldehyde fixation before running on 1×-TBE–acrylamide gels. The error bars indicate ± SEM. of three independent experiments and images are representative. (**D**) *K*_d_ and *B*_max_ values of full-length ZNF432 bound to single-strand DNA.

We have previously shown that PARP-1 affects DNA end resection of DSBs (22), this activity could also rely on PAR binding cofactors. Having established that ZNF432 stimulates PARP-1 activation, we then checked whether ZNF432 binds single-strand DNA which is produced during DNA resection. Because ZNF domains are flexible DNA-binding motifs that can recognize a broad range of structures, we monitored ZNF432 capacity to bind ssDNA versus double‐stranded DNA (dsDNA). Using radiolabeled probes corresponding to these different DNA structures, we found that ZNF432 is proficient in binding both substrates *in vitro* (Figure 6A and B) which is consistent with the fact that ZNFs bind nucleic acids structures with high versatility. However, ZNF432 binds more strongly to ssDNA (Figure 6B) and shows preferential binding to ssDNA when evaluated in a competition assay with dsDNA (Figure 6C). To reveal the relative contributions of the ZNFs/KRAB domains to DNA interactions, we purified the KRAB domain (amino acids 1–205) and ZNFs-containing domain (amino acids 205–652) (Supplemental Figure S3A). The KRAB domain displayed negligible DNA binding compared to ZNF432 and the C-terminal domain (Supplemental Figure S3B and C). The C-terminus displayed cooperative binding creating difficulties in Kd determination, while full-length ZNF432 displayed a dissociation constant of 5.41 nM on ssDNA (Figure 6D).

**Figure 7.**
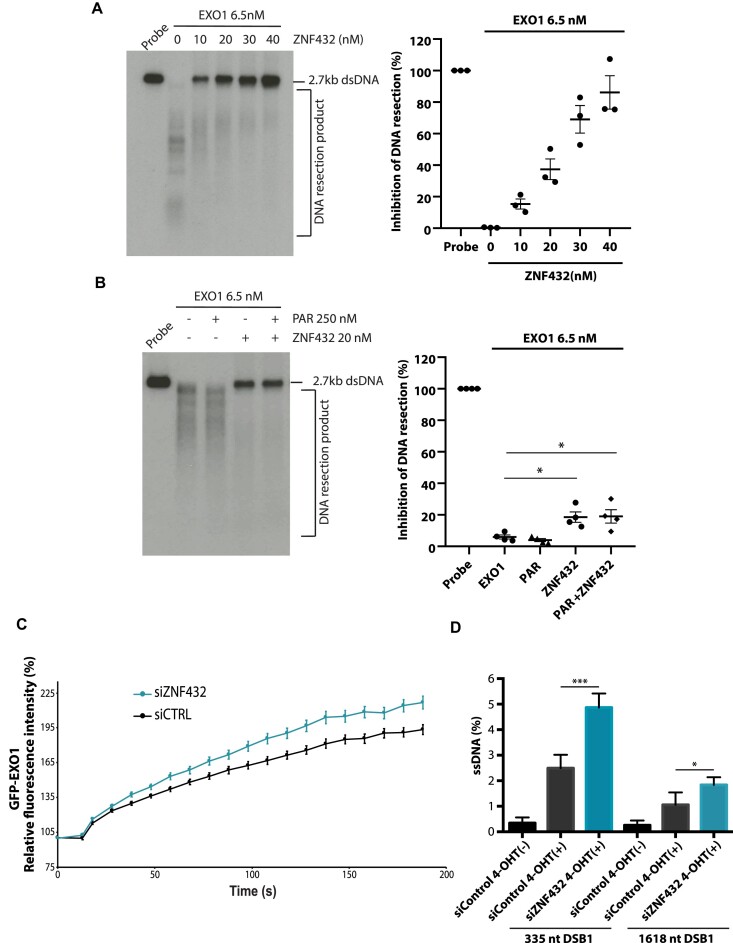
ZNF432 is an inhibitor of EXO1-mediated DNA resection. In vitro resection assay using 2.7 Kb double-strand [α-^32^P] radiolabeled DNA probe and EXO1 (6.5 nM) with (**A**) increasing concentrations of ZNF432 (0, 10, 20, 30 and 40 nM) and (**B**) with ZNF432 (20 nM) in presence or absence of 250 nM purified PAR. All images are representative of three independent biological repeats. The error bars indicate ± SEM. *P = 0.05. (**C**) Recruitment kinetics for GFP-EXO1 WT in U2OS cells transfected with siCTRL or siZNF432 to laser-induced DSBs. Mean curves ± SEM (*n* = 145) are shown. (**D**) ER-AsiS1 U2OS cells were knocked down for ZNF432 for 72 h, followed by induction with 300 nM 4-hydroxytamoxifen for 4 h and quantification of ssDNA. The error bars indicate ± s.d. from four independent experiments. *P ≤ 0.05, *** *P* ≤ 0.001 (Student's *t*-test).

These results clearly establish ZNF432 as a ssDNA-binding protein, which suggests that ZNF432 might be able to regulate DNA resection efficiency. Thus, we performed EXO1-based DNA resection assays with a 3′-end-labeled dsDNA (2.7 kb). In the absence of ZNF432, EXO1 completely resected the dsDNA substrate (Figure 7A). When the reaction was supplemented with ZNF432, concentration-dependent inhibition of DNA resection was observed. At 10 nM ZNF432, 15% of resection was inhibited while 86% was inhibited with 40 nM ZNF432 within the 30 min incubation time (Figure 7A). To determine if PAR affected the inhibitory function of ZNF432 on the EXO1-based DNA end resection machinery, we performed reactions with protein-free PAR in the presence or absence of ZNF432 (Figure 7B). The presence of PAR did not alter the inhibitory effect ZNF432 on EXO1-mediated resection. These results show that ZNF432 can robustly inhibit the DNA end resection ability of EXO1, likely through a PAR-independent DNA-binding mechanism. In support of this, microirradiation experiments showed enhanced accumulation of GFP-EXO1 in ZNF432 KD cells at DNA damage sites (Figure 7C). One prediction, based on this observation, is that ZNF432 KD cells should display enhanced formation of ssDNA at DSB sites. To quantitate ssDNA at sites of DSBs, we used the ER-*Asi*SI system in which the restriction enzyme *Asi*SI is fused to the estrogen receptor hormone-binding domain. Upon treatment with 4-OHT, the *Asi*SI nuclease translocates to the nucleus and generates up to 150 DSBs at sequence-specific sites. If a DSB has been resected, this will lead to single-stranded DNA, which cannot be cleaved by the restriction endonuclease *Bsr*GI before PCR. If DNA resection does not occur, the double-stranded DNA will be cleaved, therefore yielding no PCR products. Importantly, ZNF432 depletion led to a ∼2-fold increase in DNA resection at a DNA double-strand break compared to the control (Figure 7D). Altogether, these results show that ZNF432 limits DNA processing *in vivo*.

### Depletion of ZNF432 leads to PARPi resistance

**Figure 8.**
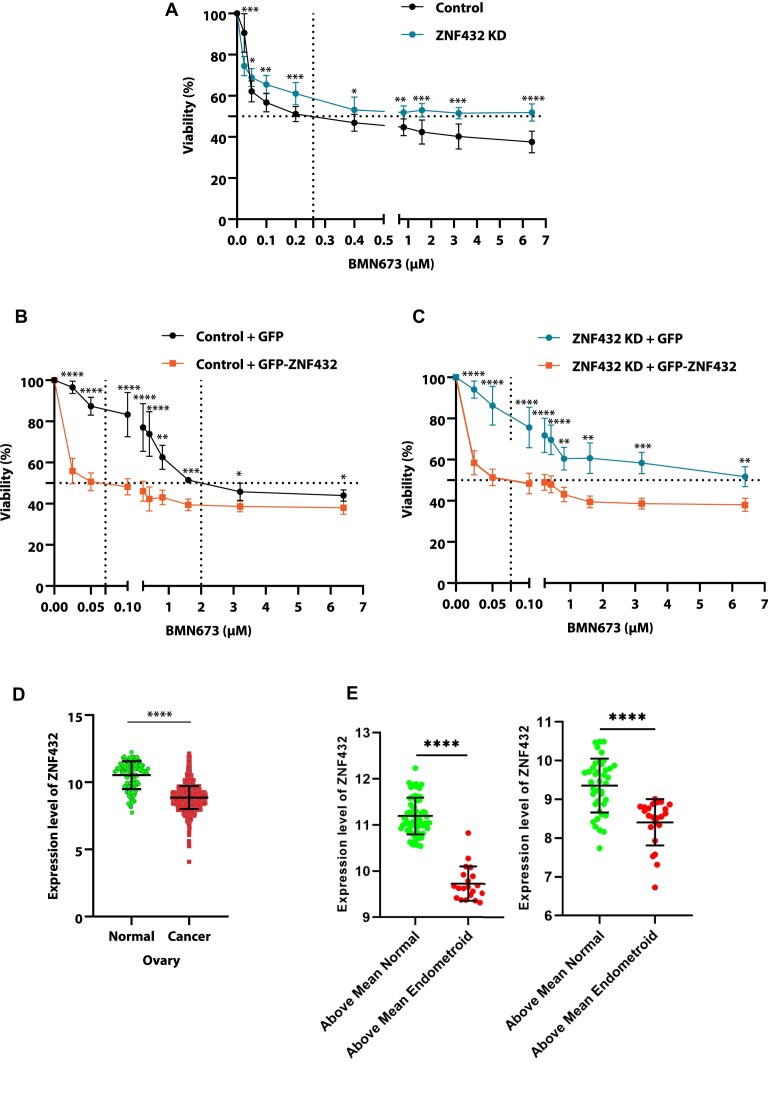
Loss of ZNF432 leads to resistance to BMN673. (**A**) Effect of BMN673 (0–6.4 μM) on survival of U2OS-Control and U2OS-ZNF432-KD cells. Effect of BMN673 (0–6.4 μM) on survival of cells overexpressing GFP or GFP-ZNF432: (**B**) U2OS-Control, (**C**) U2OS-ZNF432-KD cells. The error bar indicates ± s.d. of three independent experiments. The *P*-value was calculated by the unpaired *t*-test. (**D**) Expression of ZNF432 in ovarian normal (*n* = 110) cancer (*n* = 1516) tissue. The *P*-value was calculated by an unpaired *t*-test. (**E**) Each population (normal/endometroid) were separated based on their mean ZNF432 expression level. Comparison of the healthy population with high ZNF432 expression to the endometroid population with relatively high ZNF432 expression (left) and analysis for the lower expressing subpopulations in both groups (right). The error bar indicates ± s.d. The *P*-value was calculated by an ordinary one-way ANOVA test. The expression data was obtained from http://gent2.appex.kr/gent2/ (accessed on 24 January 2022). *P ≤ 0.05, **P ≤ 0.01, ***P ≤ 0.001, ****P ≤ 0.0001.

HR-deficient tumor cells are hypersensitive to PARPi. With this in mind, we evaluated if the restoration of HR in these cells through ZNF432 depletion could result in PARPi resistance. As a first step, we observed that attenuation of ZNF432 expression promotes resistance to PARPi in U2OS HR-proficient cells (Figure 8A). Conversely, the overexpression of GFP-ZNF432 sensitizes U2OS cells to PARPi (Figure 8B). A rescue experiment where GFP-ZNF432 is expressed in ZNF432 KD cells also restores PARPi sensitivity (Supplementary Figure S4B, Figure 8C). The difference in half maximal inhibitory concentration (IC_50_) after downregulation or overexpression of ZNF432 in the U2OS cell line is indicated in Supplementary Figure S4C. The effect of DNA damage on PARPi sensitivity was also analyzed in the U2OS cell line challenged by irradiation. The overexpression of GFP-ZNF432 led to a decreased viability (Supplementary Figure S4D). This subsequent sensitivity to PARPi following ZNF432 overexpression may be a result of the inhibition of DNA resection leading to inefficient HR and decrease in cell survival.

Considering this interesting finding, we performed gene expression analysis in ovarian cancer tissue samples and we have identified an overall lower expression of ZNF432 in ovarian cancer samples when compared to normal ovary samples (Figure 8D). Then, we divided each population (normal/endometroid) based on their mean ZNF432 expression level. We compared the healthy population with high ZNF432 expression to the endometroid population with relatively high ZNF432 expression, as depicted in Figure 8E. Similarly, we conducted an analysis for the lower-expressing subpopulations in both groups. Based on this analysis, our data clearly demonstrate that the expression of ZNF432 in endometroid ovarian cancer patients is significantly lower compared to the healthy (normal) population (high vs high and low vs low). Together with the *in cellulo* viability data, the tumour sample analysis suggests that modulation of ZNF432 expression in ovarian cancer cells could sensitize them to PARPi.

### Overexpression of ZNF432 sensitizes PARPi resistant ovarian cancer cells

**Figure 9.**
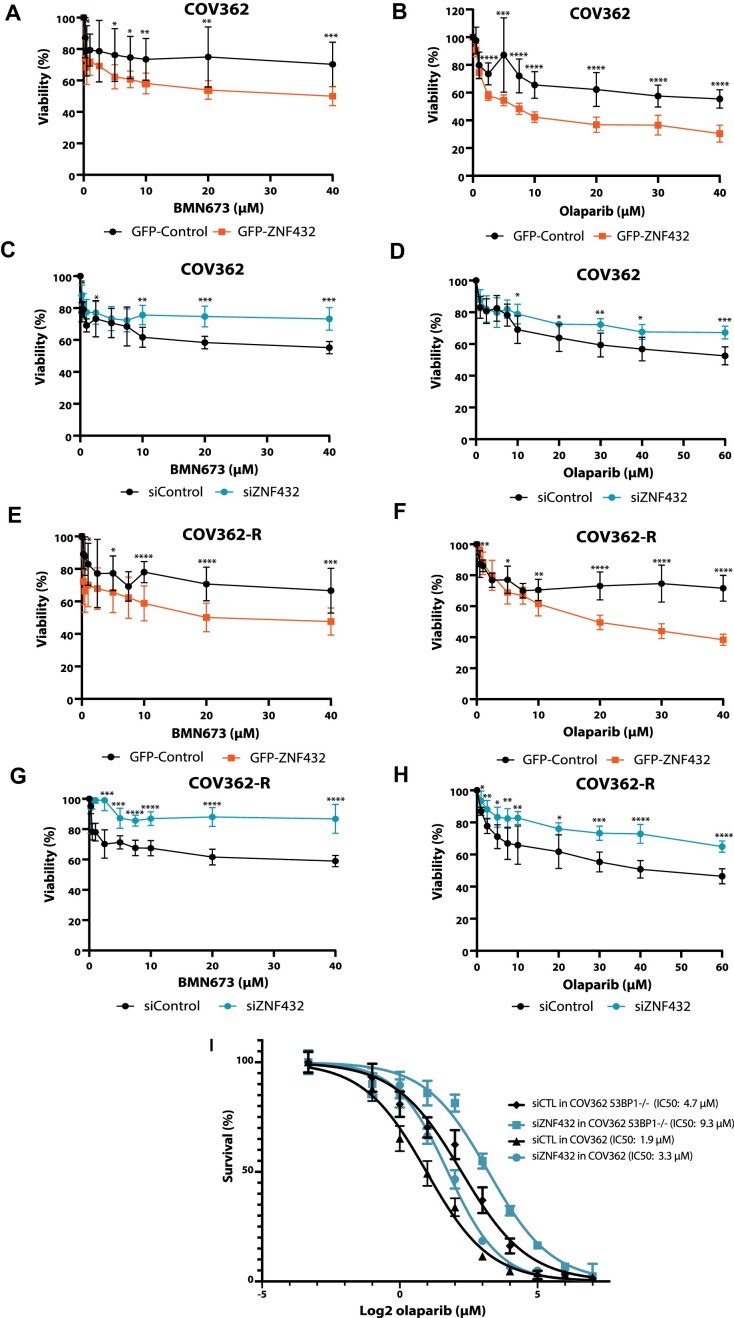
Modulation of ZNF432 sensitizes the PARPi resistant ovarian cancer cell line to PARP inhibition. Effect of BMN673 on viability following ZNF432 overexpression (**A, E**) or ZNF432 KD (**C, G**) in COV362 and COV362 Olaparib resistant (COV362-R) cells respectively. Effect of AZD2281 survival following ZNF432 overexpression (**B, F**) or ZNF432 KD (**D, H**) in COV362 and COV362-R cells. The error bars indicate ± s.d. from three independent experiments. The *P*-value was calculated by the unpaired *t*-test. *P ≤ 0.05, **P ≤ 0.01, ***P ≤ 0.001, ****P ≤ 0.0001. (**I**) Knockdown of ZNF432 in COV362 53BP1^−/−^ cells leads to additive resistance to Olaparib. The error bars indicate ± s.d. from three independent experiments.

We showed that overexpression of ZNF432 conveyed sensitivity to PARPi in U2OS cells. To assess the impact of ZNF432 overexpression in a cellular model of high-grade serous ovarian cancer, we chose the ovarian cancer cell line COV362 and an Olaparib-resistant version (COV362-R). COV362-R Olaparib-resistant cells were selected in stepwise increases of Olaparib up to 10 μM and were also shown to be cisplatin resistant due to restoration of BRCA1 expression. Hence, in contrast to the parental COV362 cell line, the Olaparib-selected subline grows in relatively high concentrations of Olaparib. We observed that overexpression of ZNF432 sensitizes COV362 and COV362-R cells to both Talazoparib (Figure 9A and E) and Olaparib (Figure 9B and F). In contrast, downregulation of ZNF432 expression by using siRNA against ZNF432 further increases their resistance to both Talazoparib (Figure 9C and G) and Olaparib (Figure 9D and H). To confirm these results in an established model of PARPi resistance, we used COV362 cell lines knockout for 53BP1 and performed live-cell proliferation phase contrast analysis using an Incucyte SX5 (Figure 9I). In this model, ZNF432 knockdown (Supplemental Figure S4E) led to 2.8-fold increased IC50 compared to COV362 53BP1 KO cells. These results demonstrate that overexpression of ZNF432 in PARPi-resistant cells sensitizes them to PARPi.

## Discussion

**Figure 10.**
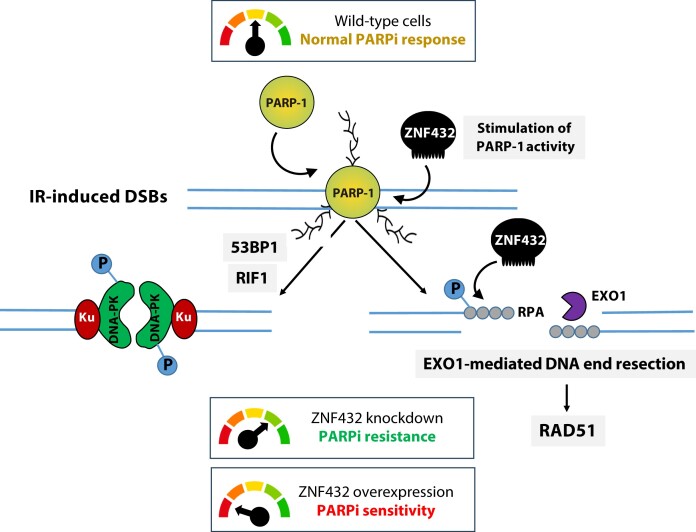
Model. In wild-type cells, PARP-1 is recruited and activated at DNA damage sites, which then engage ZNF432 to stimulate PARylation. ZNF432 promotes NHEJ and inhibits DNA end resection by binding ssDNA, although to a certain extent, so the cells can perform DNA repair. If ZNF432 is depleted, ZNF432 is no longer recruited to DSBs, it cannot activate PARP-1 and protect ssDNA, a loss of 53BP1 and RIF1 foci occurs, leading to DSB hyper-resection. This enhances PARPi resistance. Conversely, overexpression of ZNF432 downregulates DNA end resection and therefore sensitizes cells to PARPi.

In this study, we uncovered evidence of a role for ZNF432 in DDR and the maintenance of genome stability. Besides being described in 2014 as a gene appearing to influence the effect of inhaled steroids on asthma (54), there is no biological function assigned to ZNF432. Herein, we introduce ZNF432 as a novel player in the growing family of C2H2-type ZFP and KRAB domain-containing proteins involved in the regulation of DSB repair processes (55). Mechanistically, our results suggest that ZNF432 counteracts DNA DSB resection. Cells depleted of ZNF432 show a significant increase in BrdU-labeled ssDNA exposed by end resection after irradiation, which is corroborated by an increase in phosphorylated RPA and RAD51 foci formation. Using the ER*-Asi*S1 system, ZNF432 depletion led to a ∼2-fold increase in DNA resection at a DNA double-strand break compared to the control. This conclusion was further supported by the observation that ZNF432 preferentially binds ssDNA and inhibits HR *in vitro* by restricting EXO1-mediated DNA end resection. Remarkably, ZNF432 can also stimulate PARylation *in vitro* and *in cellulo*. Through these functions, the expression levels of ZNF432 finely balances PARylation and DNA end resection affecting the outcome of PARPi response (see model in Figure 10).

Although ZFPs have emerged as a new family of regulators of genomic integrity, we are far from understanding how these proteins orchestrate the DDR process, especially considering that structurally similar ZFPs can have opposite effects on the control of the DNA repair pathway choice. ZFPs that are very similar in terms of ZNFs architecture can stimulate or inhibit PARP-1 activity in an unpredictable way. For instance, ZNF432 stimulates the PARylation activity of PARP-1 *in vitro*, similar to CTCF (56) and ZBTB24 (18), as opposed to enhanced PARylation observed upon knockdown of ZNF365 (57). It remains unclear how this fine-tuning of PARP-1 activity can be coordinated by multiple ZFPs. The key to this conundrum may reside in the unique interaction profile of the C2H2-type ZFPs within their interaction network (58). KRAB ZFPs typically associate with KAP1, however, individual KRAB ZFPs also show unique and highly specific interactions. For example, the KRAB ZFPs ZFP28, ZNF273 and ZNF677 appeared to form a complex with the HR inhibitor 53BP1 (58). Our AP-MS analysis suggests a specific protein complex involving ZNF432, KAP1 and the protein SET, which might also explain the regulation of HR repair that we observed in ZNF432-depleted cells. SET is a KAP1-interacting protein that induces chromatin compaction and inhibits repair by HR (45). Similar to the PAR-regulated chromatin remodeler ALC1, ZNF432 might play a role in the mobilization of PARP-1 from the DNA lesions (59). Recently, we have shown that PARP-1 activity antagonizes the DNA end resection machinery which is a critical step for directing repair toward HR (22). One possibility would be that ZNF432, KAP1, and SET act together to moderate PARP-1-induced chromatin relaxation. This idea is supported by the observation that knockdown of the C2H2-type ZFP ZNF668 impairs chromatin relaxation, causes decreased recruitment of repair proteins to DNA lesions and correlates with defective HR repair (60). Therefore, the complex interplay between ZFPs, PARP-1, and PAR polymers might be decisive in dictating the DNA repair pathway choice. Certainly, the ZNF432 C-terminal region containing the 16 ZNFs is a key determinant of PARP-1 stimulation of PARylation, as the KRAB domain was inefficient in PARP-1 PARylation assays.

For ZFPs with long arrays of ZNFs, such as ZNF432, it appears as though not all the ZNF repeats are engaged in DNA recognition (61). As evidence accumulates, it is very likely that some of these extra fingers possess a significant affinity for PAR polymers as seen for CTCF (56), ZBTB24 (19), or E4F1 (20). The identification of several ZFPs as specific *readers* of PAR suggests that the affinity of these proteins for PAR might be a widespread feature applicable to multiple members of the C2H2-type family of proteins (23,62), including ZNF432 for which we observed significant PAR-binding activity *in vitro*. A simulation study has shown that the affinity of ZFPs to a specific DNA target is larger than the sum of affinities of individual fingers and depends on multiple factors such as specific site and linker length between ZNFs (63). How PAR binding contributes to the affinity and selectivity of ZFPs at DNA damage sites remains to be elucidated.

We have previously shown that PARylation can recruit the ATM kinase to sites of DNA damage (64) and many chromatin remodelling proteins have also been shown to be recruited in a PARP-dependent manner, driving chromatin decondensation (recently reviewed in (65,66)). Collectively, the results suggest that, by influencing PARP-1 chromatin binding and catalytic activity, ZNF432 may influence the efficiency of early upstream signaling through effects downstream of PARP-1 recruitment and activation. PARylation may promote further assembly of PARP-1 on the chromatin by further increasing accessibility. These effects were modest compared to the changes in RAD51 assembly, where the impact is pronounced.

We have used RAD51 foci and phospho-DNA-PKcs foci as markers of HR and NHEJ respectively at irradiated sites. The dose we used is 5 Gy (∼180 DSBs/genome) and the number of foci is representative of HR/NHEJ which repair DSBs at different sites in the genome. Most likely, these sites have different chromatin environments and are located at different loci undergoing transcription/replication or not. Hence, using DNA repair cassettes might not be totally accurate to represent this complexity, as DNA repair is measured in a single locus. While a reduction of NHEJ was observed, we did not see significant HR difference at a CRISPR/LMNA single locus. Further studies on ZNF432 will involve monitoring HR at different loci.

Overall, our results converge on the conclusion that ZNF432 counteracts EXO1-mediated DNA end resection, through its ZNFs repeat, unlike the KRAB domain, as they tightly bound ssDNA. In addition, GFP-EXO1 was localized more abundantly in ZNF432 KD cells at DNA damage sites. Negative HR regulators that prevent ssDNA over-accumulation have recently gained attention for their propensity to promote PARPi resistance when depleted or inactivated (26). We found that ZNF432 knockdown leads to the reduction of 53BP1 and RIF1 foci following irradiation, two resection repressors. The decrease of pDNA-PKcs foci that we observed after ZNF432 depletion is also consistent with the fact that ZNF432 expression negatively regulates RAD51 foci formation. Since HR restoration is frequently observed in PARPi resistant tumors (67), we presumed that loss of ZNF432 would confer PARPi resistance in HR-deficient cell lines. Our data confirm our assumptions, where we show that downregulation of ZNF432 in U2OS and ovarian cancer cells leads to resistance to PARPi, whereas over-expression rescues this phenotype. The classical route of HR restoration and PARPi resistance in a BRCA1-deficient background occurs via reactivation of DNA resection by deregulation of 53BP1 (68,69), RIF1 (70), or DYNLL1 (71). Therefore we used COV362 53BP1 KO cells, an established model of PARPi resistance, to monitor the effect of ZNF432. In this model, we observed that KD of ZNF432 led to 2.8-fold increased resistance compared to COV362 53BP1 KO cells. Thus, removing two DNA resection inhibitors (53BP1 and ZNF432) leads to additive PARPi resistance. Conversely, the effect of overexpression of ZNF432 on PARPi sensitization is mediated by inhibition of DNA resection thereby sensitizing to PARPi.

In summary, while introducing a new player in the DDR field, the present study widens the avenue of PARPi efficacy beyond the realms of HR-deficient cancer subtypes. Identifying effective biomarkers to predict the response to PARPis for precision medicine is paramount. Herein, we show that the levels of ZNF432 should be closely monitored in tumors to predict PARPi efficacy. In addition to ZNF432, an ever-increasing number of ZFPs have been shown to impact genome stability which could eventually lead to the establishment of a predictive ZFP gene signature for PARPi response with prognostic value in ovarian cancer.

## Supplementary Material

gkad791_Supplemental_Files

## Data Availability

We used the GENT2 (Gene expression database of Normal and Tumor tissues) open platform for gene expression data covering different normal and tumor tissue sample data from public gene expression data sets (http://gent2.appex.kr/gent2/). The mass spectrometry proteomics data have been deposited to the ProteomeXchange Consortium (http://www.proteomexchange.org) via the PRIDE partner repository with the dataset identifier PXD038731. The data underlying this article will be shared on reasonable request to the corresponding author.
