# Supplementary material for: ZNF432 stimulates PARylation and inhibits DNA resection to balance PARPi sensitivity and resistance
Source: Nucleic Acids Res. 2023 Oct 12;51(20):11056–79. doi: 10.1093/nar/gkad791 (PMC10639050; doi:10.1093/nar/gkad791)
Supplement: gkad791_Supplemental_Files [file gkad791_supplemental_files.zip › _ Supplementary data ZNF432 V11 FINAL.pdf]

ZNF432 stimulates PARylation and inhibits DNA resection to balance PARPi sensitivity and resistance

Julia O'Sullivan, Charu Kothari, Marie-Christine Caron, Jean-Philippe Gagné, Zhigang Jin, Louis Nonfoux, Adèle Beneyton, Yan Coulombe, Mélissa Thomas, Nurgul Atalay, X Wei Meng, Larissa Milano, Dominique Jean, François-Michel Boisvert, Scott H. Kaufmann, Michael Hendzel, Jean-Yves Masson*, and Guy G. Poirier**

*Co-corresponding authors

Supplementary Figure legends

Supplementary Figure 1:

ZNF432 inhibition enhances HR-mediated DSB repair.

(**A**) ZNF432 knockdown efficiency in HeLa, EJ5-GFP HEK293T, ER-*Asi*S1 U2OS cells.

(**B**) Western blot showing the expression level of ZNF432 in U2OS wild-type (U2OS-Control) and ZNF432 knock-down clones (C5 and F1). For survival analysis, only the F1 clone was selected and is referred to as U2OS ZNF432 KD.

Effect of siZNF432 on RAD51 foci formation post-IR induced DNA damage in HeLa cells (**C**) or in U2OS CRISPR-Cas generated ZNF432 KD cells (**D**). Effect of ZNF432 on BrdU foci formation post-IR induced damage in CRISPR-Cas generated ZNF432 KD cells as compared to control cells (**E**). The graphs are representative of three independent experiments and error bars indicate ± s.d. The p-value is calculated by the Mann-Whitney test. * ≤ 0.05, ** ≤ 0.01, *** ≤ 0.001, **** ≤ 0.0001.

Supplementary Figure 2: Downregulation of ZNF432 by CRISPR-CAS KD decreases 53BP1 foci (**A**) and RIF1 foci (**B**) formation upon IR-induced DNA damage.

(**C**) Downregulation of ZNF432 by siRNA against ZNF432 shifts the cell cycle to the G2/M phase in HeLa cells. The error bars indicate ±s.d. * ≤ 0.05, ** ≤ 0.01, *** ≤ 0.001, **** ≤ 0.0001.

Supplementary Figure 3: (**A**) SDS-PAGE of purified full-length ZNF432, the KRAB domain (amino acids 1-205) or ZNF-containing domain (amino acids 205-652). (**B**) Single-strand DNA binding assays with the indicated concentrations of proteins. (**C**) Quantification of the percentage of ssDNA binding in (B) from experiments done in

triplicate. The background values in the mock control were removed from the quantification.

Supplementary Figure 4:

(A) A violin plot displaying the quantification of relative PAR intensity in the whole nucleus of control (n=104) or ZNF432 KD (n=151) cells. The dotted line indicates the mean. The slide line below the mean labels the 1st quartile and the slide line above the mean demarcate the 3rd quartile, respectively.

(B) Western blot showing the endogenous protein level of ZNF432 (75kDa) in U2OS and ZNF432 F1 clone and overexpression of GFP-ZNF432 (100 KDa).

(C) The difference in IC50 of BMN673 upon downregulation or upregulation of ZNF432 in the U2OS cell line. The error bars indicate ±s.d.

(**D**) The viability of irradiated U2OS cells (5 Gy) is reduced upon overexpression of GFP-ZNF432. The error bars indicate ±s.d.

(**E**) Effect of siRNA against ZNF432 on its expression level in COV362 and COV362 53BP1$^{-/-}$ cell line. ZNF432 was detected by western blotting and protein loading was monitored by Ponceau staining.

Supplementary Table 1: Antibodies used in this study

Antibodies	Source	Dilution used	Catalog number
Geminin	Abcam	1 :5000 IF	ab104306
Geminin	Proteintech	1 :300 IF	10802-1-AP
RPA2	Abcam	1 :1000 IF	ab2175
RPA32/RPA2 (phospho S4 +S8	Abcam	1 :500 IF	Ab87277
Alpha-tubulin	Abcam	10,000 WB	ab7291
pDNA-PKcs - S2056	Abcam	1 :1000 IF	ab18192
53BP1	Novus Biologicals	1 :500 IF	NB100-304
ZNF432	Novus Biologicals	1 :1000 WB	NBP2-31813
PCNA	Novus Biologicals	1 :1000 IF	NBP267390
GFP	Roche	1 :5000 WB	11814460001
Vinculin	Millipore Sigma	1 :100000 WB	V9131
RAD51	Bioacademia	1 :5000 IF	70-001
BrdU	GE Healthcare	1 :1000 IF	RPN202
PAR	Tulip Biolabs	1 :5000 WB	1020
Rabbit anti-PAR	In-house	1 :1000 IF	96-10
γ-H2AX	Active motif	1 :2000 IF	39117
RIF1	Bethyl Laboratories	1 :400 IF	A300-569A
PARP-1	In-house	1 :5000 WB<br>1 :1000 IF	clone C2-10
Peroxidase AffiniPure Goat Anti-Rabbit IgG	Jackson Immunoresearch Laboratories	1 :5000 WB	111-035-144
Peroxidase AffiniPure Goat Anti-Mouse IgG	Jackson Immunoresearch Laboratories	1 :5000 WB	115-035-146
Alexa Fluor 488 goat antirabbit	Molecular probes	1 :1000 IF	A11008
Alexa Fluor 488 goat anti-mouse	Molecular probes	1:1000 IF	A11001
Alexa 568 goat anti-mouse	Molecular probes	1:1000 IF	A11004
Alexa 568 goat anti-rabbit	Molecular probes	1 :1000 IF	A11011

IF : Immunofluroscence. WB : Western blotting

Supplementary Table 2: Key reagents and resources

Reagent	Source	Catalog number
pcDNA3.1-ZNF432(NM_014650.2)-DYK	GenScript	OHu20285D
pRP[Exp]-CMV>EGFP/ZNF432-ZNFs (206-652)	VectorBuilder Inc.	
pRP[Exp]-CMV>EGFP/ZNF432-KRAB(1-205)	VectorBuilder Inc.	
Dulbecco's modified Eagle's medium (DMEM)	Wisent	319-165-CL
Penicillin-streptomycin solution	Wisent	450-200-EL
Phosphate buffered saline (PBS)	Wisent	311-010-CL
RPMI 1640	Wisent	350-002-CL
Hyclone fetal bovine serum	ThermoFisher Scientific	SH30070.01
Charcoal striped serum	Sigma	F6765
Opti-MEM™-reduced serum medium	ThermoFisher Scientific	31985062
Gibco™ Grace's Insect Medium	ThermoFisher Scientific	11605094
Gibco™ Sf-900™ II SFM	ThermoFisher Scientific	10902088
ProLong™ Gold Antifade Mountant	ThermoFisher Scientific	P10144
Pierce C18 Tips	ThermoFisher Scientific	87782
Invitrogen Dynabeads™ Protein G	ThermoFisher Scientific	10003D
Invitrogen Lipofectamine™ 2000 transfection reagent	ThermoFisher Scientific	11668019
Invitrogen Lipofectamine™ RNAiMAX transfection reagent	ThermoFisher Scientific	13778075
TransIT-293 Transfection Reagent	Mirus	MIR2700
Effectene™ transfection reagent	Qiagen	301425
Plasmid Maxi Kit	Qiagen	12162
Trypsin/Lys-C Mix	Promega Corporation	V5071
Bovine serum albumin	Millipore Sigma	9048-46-8
BrDU	Millipore Sigma	B5002
Hoechst 33342	Millipore Sigma	14533
DAPI	Millipore Sigma	D9542
Talazoparib/BMN-673	Selleckchem	S7048
AlamarBlue®	Bio-Rad	BUF012A
0.2 µm pore size nitrocellulose membrane	Bio-Rad	1620112
SYPRO® Ruby Protein Gel Stain	Bio-Rad	1703125
Precision Plus™ Protein Dual Color Standards	Bio-Rad	1610374
Purified PARP-1	Tulip Biolabs	2090
HPF1	Tulip Biolabs	2097
DNA oligonucleotides	Integrated DNA Technologies	
Western Lightning® Plus ECL chemiluminescence substrate	PerkinElmer	NEL103E001EA
[α-^{32}P]-ATP	PerkinElmer	NEG512H250UC
Terminal transferase	New England Biolabs	M03155
DNA-dependent protein kinase (DNA-PK) inhibitor NU7741	Selleck Chemicals	S2638
4-Hydroxytamoxifen	Sigma	H7904-5MG

BsrG1 HF	New England Biolabs	R3575S
Applied Biosystems PowerUp SYBR Green Master Mix	ThermoFisher Scientific	A25742
RNase H	New England Biolabs	M0297L

Supplementary Table 3: siRNA sequences used in this study

Pool Catalog Number	Duplex Catalog Number	Gene Symbol	GENE ID	Gene Accession	GI Number	Sequence
**L-015224-02**	J-015224-17	ZNF354B	117608	NM_058230	2.07E+08	CUUAAUAGACACCGAAUAA
	J-015224-18	ZNF354B	117608	NM_058230	2.07E+08	GCUUGAGAGUGAUUUAUUA
	J-015224-19	ZNF354B	117608	NM_058230	2.07E+08	AUGUAAGAGCACACCUAAA
	J-015224-20	ZNF354B	117608	NM_058230	2.07E+08	UGUAGAUAGAAGCCAUAAA
**L-020424-02**	J-020424-17	ZNF510	22869	NM_014930	7662423	AGUCAACUGUGGAGGAAUA
	J-020424-18	ZNF510	22869	NM_014930	7662423	GGAAGAAAUUUGUCCGGAA
	J-020424-19	ZNF510	22869	NM_014930	7662423	GAAAAUAGGUUGCGGUAAU
	J-020424-20	ZNF510	22869	NM_014930	7662423	AGUCAUACCCUAUUAGAUA
**L-020326-01**	J-020326-09	ZNF432	9668	NM_014650	45331209	GAUGAAAGGCACAGUCGAA
	J-020326-10	ZNF432	9668	NM_014650	45331209	CAUCAUACAUCAACGAAAU
	J-020326-11	ZNF432	9668	NM_014650	45331209	CUAAAUCCCAAGUCAGUAA
	J-020326-12	ZNF432	9668	NM_014650	45331209	GGAAGAGCAUGCUUAUUAU
**L-013946-02**	J-013946-17	ZNF385A	25946	NM_001130968	1.96E+08	CCGACGAGUCAAAGGCAUU
	J-013946-18	ZNF385A	25946	NM_001130968	1.96E+08	CUACAAAGGUAAUCGCCAC
	J-013946-19	ZNF385A	25946	NM_001130968	1.96E+08	UGUGAGAUCUGCAAUGUCA
	J-013946-20	ZNF385A	25946	NM_001130968	1.96E+08	GAUCCAAGUUCCUAGACCU
**L-012775-02**	J-012775-17	ZNF32	7580	NM_006973	53759139	GCUUCAGGAAUCAGAGUAA
	J-012775-18	ZNF32	7580	NM_006973	53759139	UAACGUUACAUGAGAGAAU
	J-012775-19	ZNF32	7580	NM_006973	53759139	UGUUACACAUCAACGGAUA
	J-012775-20	ZNF32	7580	NM_006973	53759139	CUGAAGCCCACCACAAAUA
**L-024395-02**	J-024395-17	ZNF608	57507	NM_020747	1.54E+08	CAACAUGACGGCUGCGUUA
	J-024395-18	ZNF608	57507	NM_020747	1.54E+08	GAGGAGAGCCACAGGCGAA
	J-024395-19	ZNF608	57507	NM_020747	1.54E+08	CCUCAAUGCCAGCGGACGA
	J-024395-20	ZNF608	57507	NM_020747	1.54E+08	GCAAAUGGACUGUCGGAGU
**L-017041-01**	J-017041-13	ZNF433	163059	NM_001080411	1.23E+08	CGUAUGAAUGUAAGGGUUA
	J-017041-14	ZNF433	163059	NM_001080411	1.23E+08	ACUCUAUGUUUGUGAGGAA
	J-017041-15	ZNF433	163059	NM_001080411	1.23E+08	GGUCAUCAGCAUGGAGAAA
	J-017041-16	ZNF433	163059	NM_001080411	1.23E+08	AGGAGAAACCUAAGAAUUG
**D-001810-10**	D-001810-01	Control	None	None	None	UGGUUUACAUGUCGACUAA
	D-001810-02	Control	None	None	None	UGGUUUACAUGUUGUGUGA
	D-001810-03	Control	None	None	None	UGGUUUACAUGUUUUCUGA
	D-001810-04	Control	None	None	None	UGGUUUACAUGUUUUCCUA
**siControl**		None	None	None	None	UUCGAACGUGUCACGUCAA

Supplementary Table 4: Sequence of γ-^{32}P-labeled DNA oligonucleotides

Name	Sequence
Single-stranded DNA	5'GGGCGAATTGGGCCCGACGTCGCATGCTCCTCTAGACTCGAGGAATTCG GTACCCCGGGTTCGAAATCGATAAGCTTACAGTCTCCATTTAAAGGACAAG
Double-stranded DNA	5'GGGCGAATTGGGCCCGACGTCGCATGCTCCTCTAGACTCGAGGAATTCG GTACCCCGGGTTCGAAATCGATAAGCTTACAGTCTCCATTTAAAGGACAAG<br><br>5'CTTGTCCTTTAAATGGAGACTGTAAGCTTATCGATTTCGAACCCGGGGTA CCGAATTCCTCGAGTCTAGAGGAGCATGCGACGTCGGGCCCAATTCGCCC
5'-overhang DNA	5'GGGCGAATTGGGCCCGACGTCGCATGCTCCTCTAGACTCGAGGAATTCG GTACCCCGGGTTCGAAATCGATAAGCTTACAGTCTCCATTTAAAGGACAAG<br><br>5'-CTTGTCCTTTAAATGGAGACTGTAAGCTTATCGATTTCGAACCCGGGGTA
3'-overhang DNA	5'GGGCGAATTGGGCCCGACGTCGCATGCTCCTCTAGACTCGAGGAATTCG GTACCCCGGGTTCGAAATCGATAAGCTTACAGTCTCCATTTAAAGGACAAG<br><br>5'CCGAATTCCTCGAGTCTAGAGGAGCATGCGACGTCGGGCCCAATTCGCC C

Supplementary Table 5: qPCR primer sequences used in ER-*Asi*SI resection assay.

Name	Sequence 5'-3'
DSB-335 FW	GAATCGGATGTATGCGACTGATC
DSB-335 REV	TTCCAAAGTTATTCCAACCCGAT
DSB-1618 FW	TGAGGAGGTGACATTAGAACTCAGA
DSB-1618 REV	AGGACTCACTTACACGGCCTTT

Supplementary Table 6: Mass spectrometry data of ZNF432 expression in HEK293T wildtype and PARP-1 KO cells.

The table is attached in a separate excel sheet and available in ProteomeXchange accession number: PXD038731.
