# Supplementary figures and images for: ZNF432 stimulates PARylation and inhibits DNA resection to balance PARPi sensitivity and resistance
Source: Nucleic Acids Res. 2023 Oct 12;51(20):11056–79. doi: 10.1093/nar/gkad791 (PMC10639050; doi:10.1093/nar/gkad791)

# Supplemental Figure 1

**A**

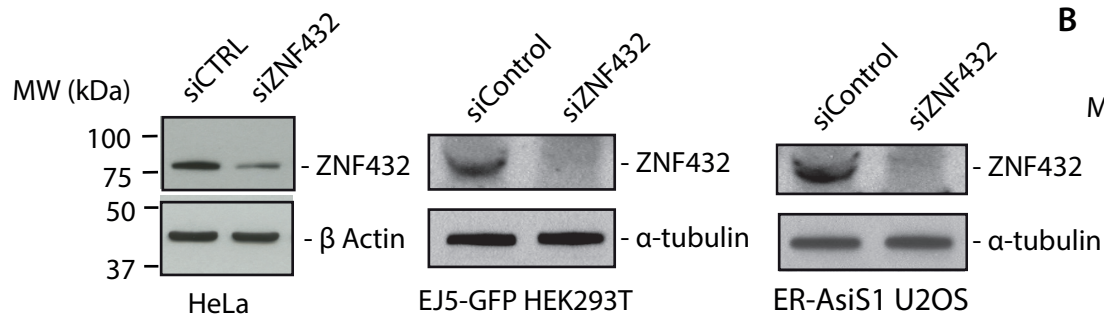

**B**

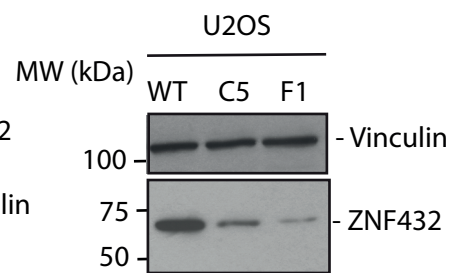

**C**

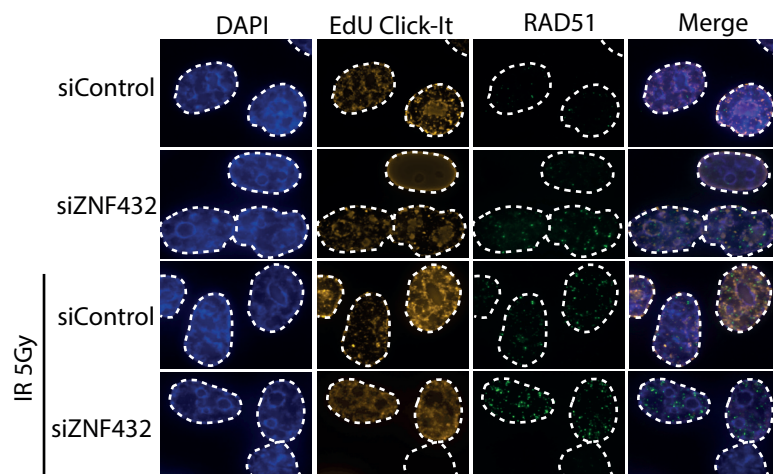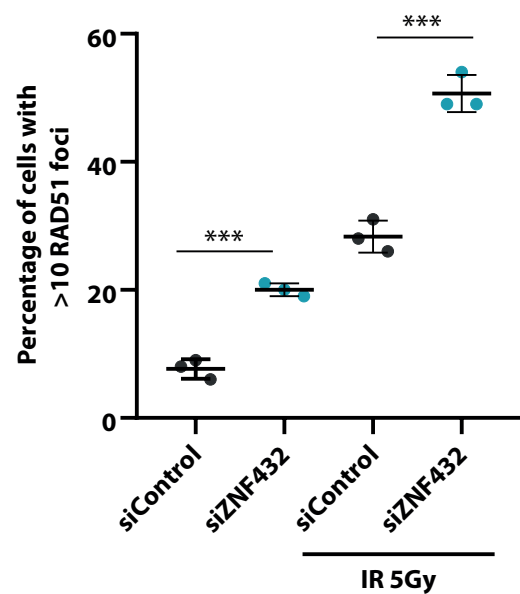

**D**

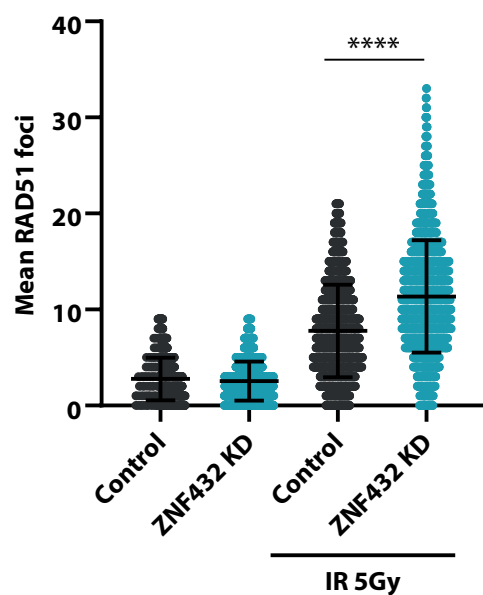

**E**

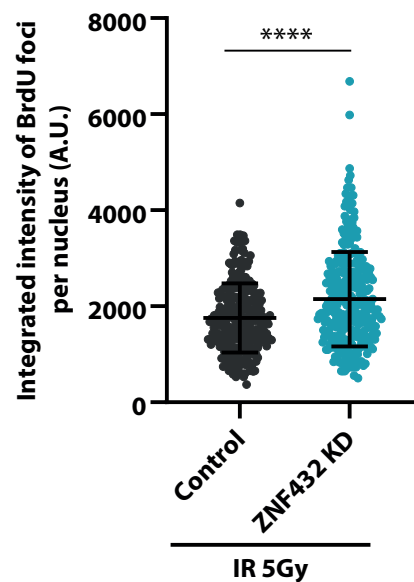

Supplemental Figure 2

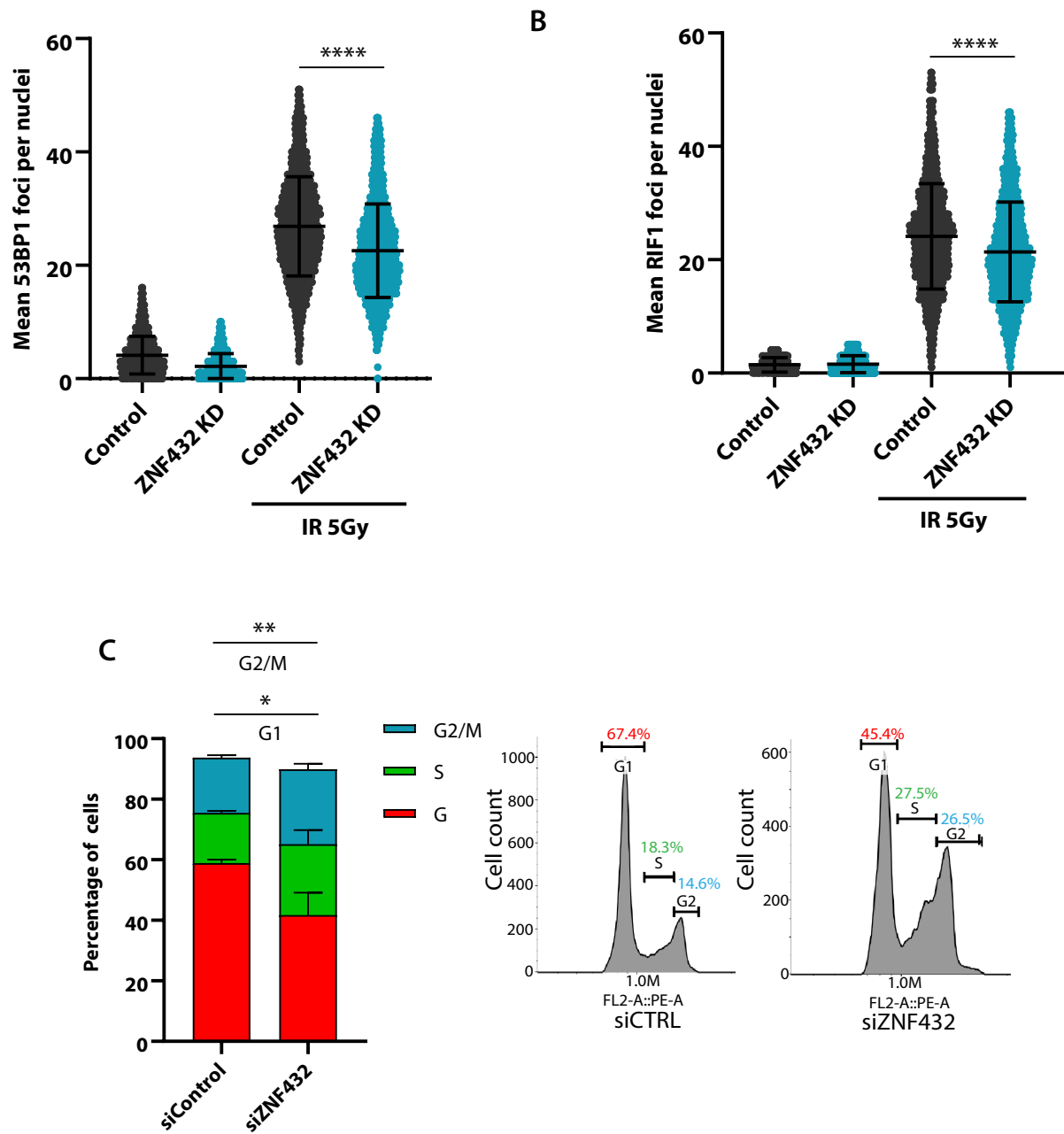

Supplemental Figure 3

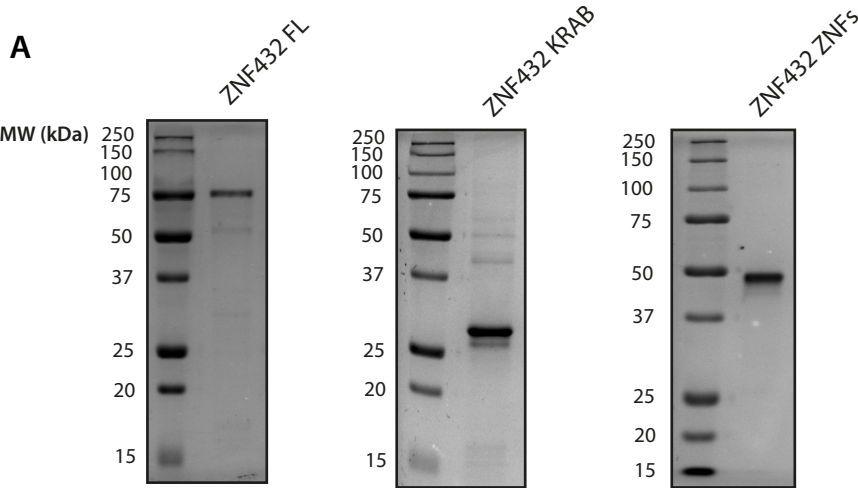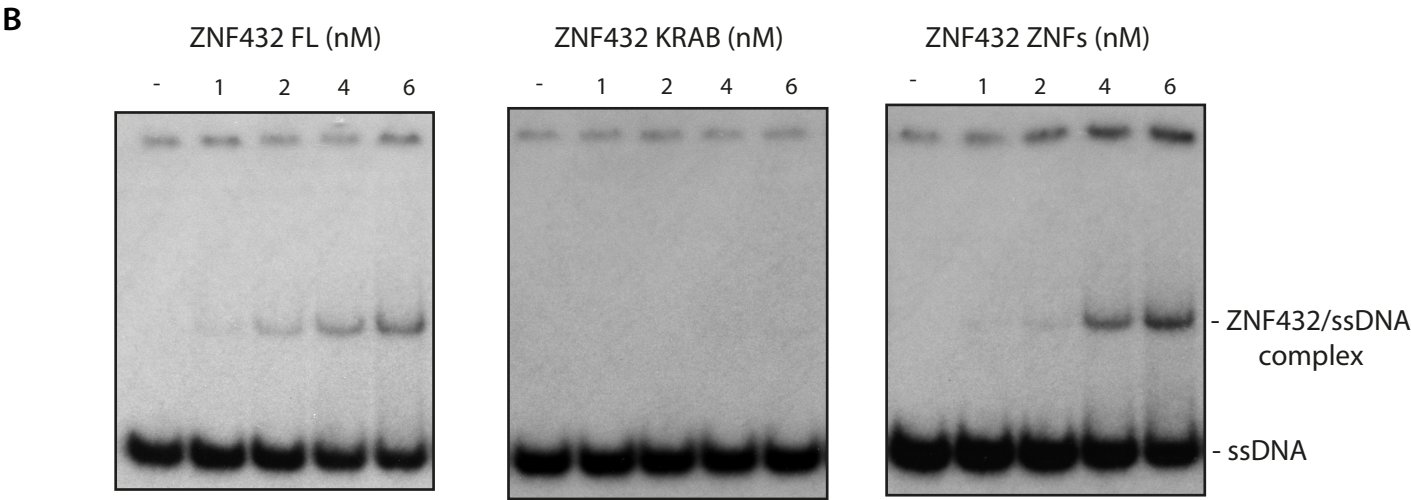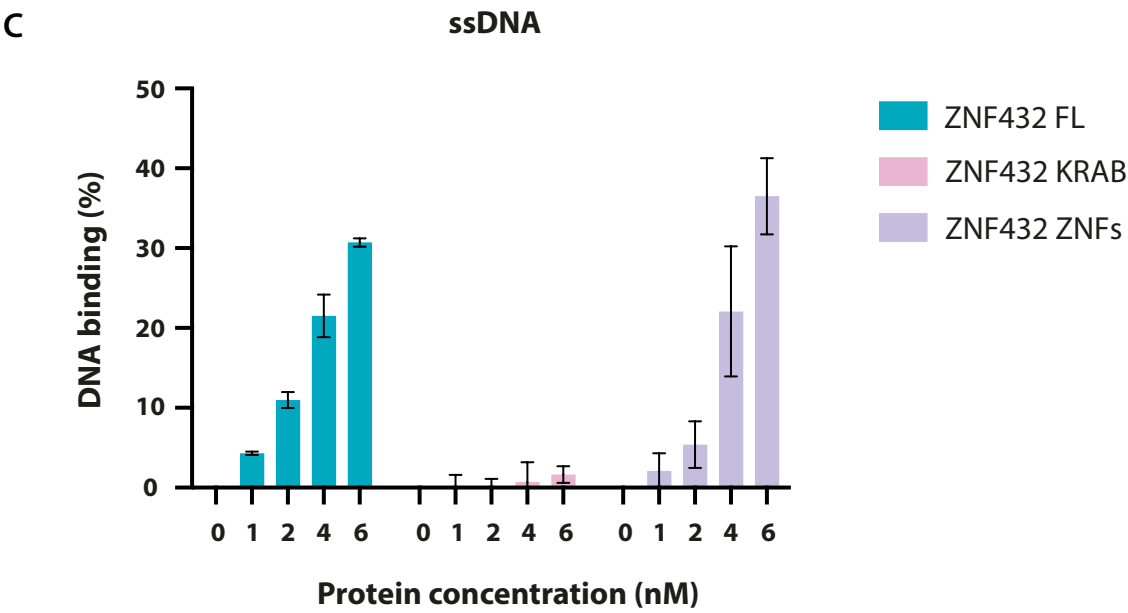

Supplemental Figure 4

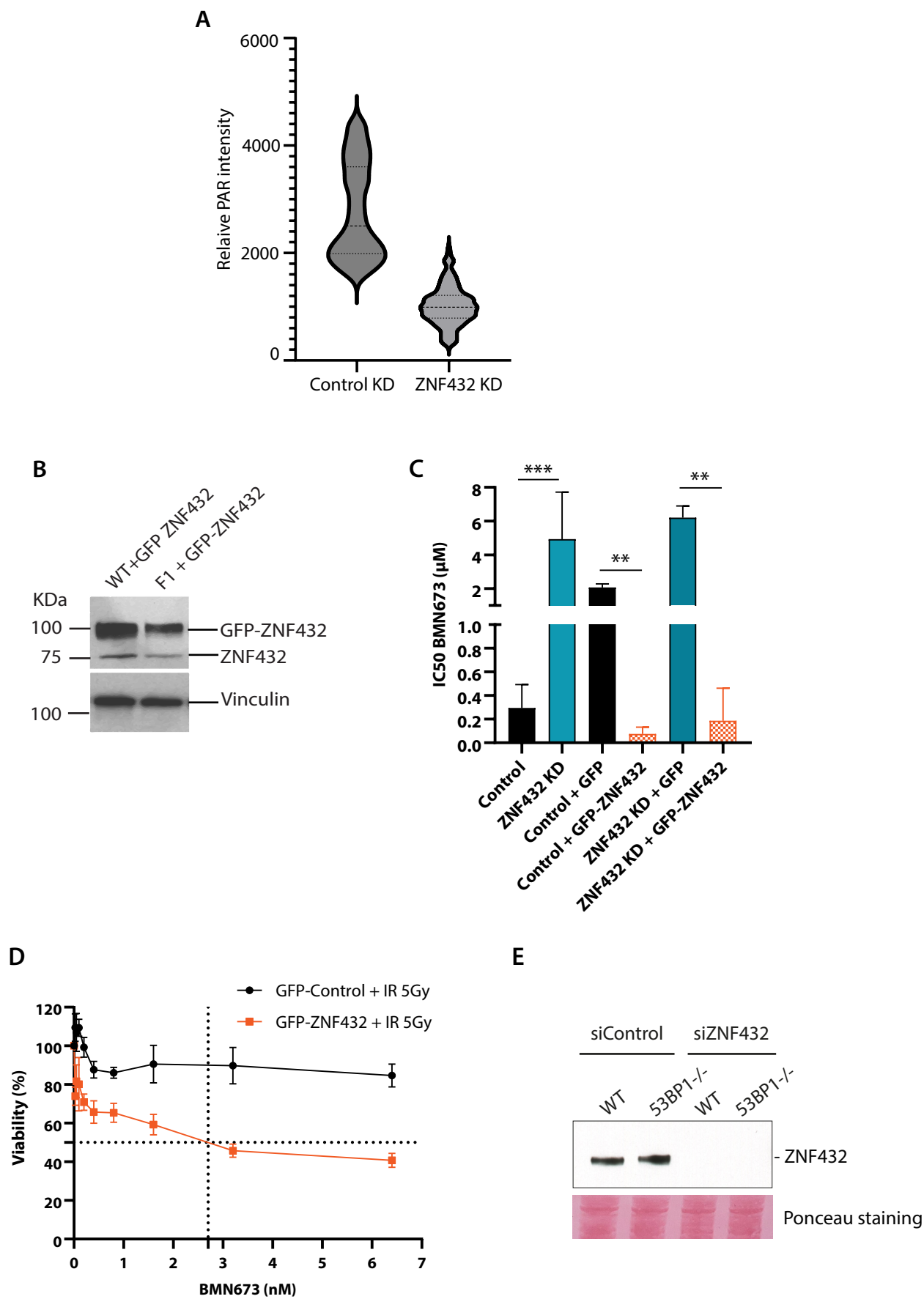

Supplement: gkad791_Supplemental_Files [file gkad791_supplemental_files.zip › _ ZNF432 Figures_V11 ONLY SUPP.pdf]
